# Land Use Change and Water Quality Use for Irrigation Alters Drylands Soil Fungal Community in the Mezquital Valley, Mexico

**DOI:** 10.3389/fmicb.2019.01220

**Published:** 2019-06-14

**Authors:** Kathia Lüneberg, Dominik Schneider, Nicole Brinkmann, Christina Siebe, Rolf Daniel

**Affiliations:** ^1^Departamento de Ciencias Ambientales y del Suelo, Instituto de Geología, Universidad Nacional Autónoma de México, Ciudad de México, Mexico; ^2^Genomic and Applied Microbiology and Göttingen Genomics Laboratory, Institute of Microbiology and Genetics, Universität Göttingen, Göttingen, Germany; ^3^Forest Botany and Tree Physiology, Büsgen-Institut, Universität Göttingen, Göttingen, Germany

**Keywords:** dryland agriculture, wastewater, fungal communities, fungal guilds, shrubland

## Abstract

Soil fungal communities provide important ecosystem services, however, some soil borne representatives damage agricultural productivity. Composition under land-use change scenarios, especially in drylands, is rarely studied. Here, the soil fungal community composition and diversity of natural shrubland was analyzed and compared with agricultural systems irrigated with different water quality, namely rain, fresh water, dam-stored, and untreated wastewater. Superficial soil samples were collected during the dry and rainy seasons. Amplicon-based sequencing of the ITS2 region was performed on total DNA extractions and used the amplicon sequence variants to predict specific fungal trophic modes with FUNGuild. Additionally, we screened for potential pathogens of crops and humans and assessed potential risks. Fungal diversity and richness were highest in shrubland and least in the wastewater-irrigated soil. Soil moisture together with soil pH and exchangeable sodium were the strongest drivers of the fungal community. The abundance of saprophytic fungi remained constant among the land use systems, while symbiotic and pathogenic fungi of plants and animals had the lowest abundance in soil irrigated with untreated wastewater. We found lineage-specific adaptations to each land use system: fungal families associated to shrubland, rainfed and part of the freshwater were adapted to drought, hence sensitive to exchangeable sodium content and most of them to N and P content. Taxa associated to freshwater, dam wastewater and untreated wastewater irrigated systems show the opposite trend. Additionally, we identified potentially harmful human pathogens that might be a health risk for the population.

## Introduction

Studies of soil fungal communities are increasing due to their ecological importance. Fungi are the major eukaryotic lineage in terms of biomass in soil, surpassing all other soil organisms combined (excluding plant roots) (Orgiazzi et al., 2012). Saprotrophic fungi participate in critical processes, such as the decomposition and mineralization of both recalcitrant and labile compounds of plant and animal origin, such as cellulose, hemicellulose, lignin, and chitin (Treseder and Lennon, 2015). Fungi are also involved in symbiosis with plant roots [arbuscular mycorrhiza (AMF) and ectomycorrhiza (EcM)], which enable plants to survive in water and nutrient limited environments (Bennett et al., 2017). Fungal communities are also known to include soil-born pathogenic representatives (Tedersoo et al., 2014). Despite their importance in maintaining soil quality and ecosystem functioning, the fungal community composition in soils is less studied than the bacterial part (Tedersoo et al., 2014). The structure and diversity of soil fungal communities are driven by soil physicochemical characteristics, such as moisture (controlled by precipitation or irrigation) (Tedersoo et al., 2014; Zheng et al., 2017), organic C availability (Lauber et al., 2008), and nutrient content (He et al., 2016) as well as the plant community (Yang et al., 2017).

Drylands are regions with arid, semi-arid, or dry sub-humid climate (Koohafkan and Stewart, 2008). This type of ecosystem covers 40% of Eartht’s surface and it is likely to expand due to climate change (Önder et al., 2009; Maestre et al., 2015). The agricultural productivity in drylands, although limited by soil moisture, supports one third of the global population (United Nations Environmental Management Group [UNEMG], 2011). As freshwater resources are scarce in these regions, it is common to use treated and untreated wastewater for irrigation to enhance the productivity (Siebe, 1998; United Nations Environmental Management Group [UNEMG], 2011; Norton-Brandão et al., 2013; Becerra-Castro et al., 2015).

The land cover change from shrubland to agriculture in drylands often diminishes soil carbon stocks and modifies the quantity and quality of organic matter, especially if it is converted to maize monoculture (Sánchez-González et al., 2017). The use of wastewater for crop irrigation particularly improves the availability of labile organic carbon and nutrients (N and P) in soil (Siebe, 1998), but it also increases soil carbon stocks due to large root residue inputs (Sánchez-González et al., 2017). However, this practice adds soluble salts, heavy metals, pharmaceuticals, and microbial pathogens to the soil, which might affect the microbial soil communities, as well as crop and human health (Siebe, 1995, 1998; Hong and Moorman, 2005; Broszat et al., 2014; Dalkmann et al., 2012).

Soil fungal communities affected by land use change and management have been frequently studied in forest and grassland ecosystems, often contrasting between tilled or fertilized systems (Birkhofer et al., 2012; Klabi et al., 2015; He et al., 2016; Wang et al., 2016). Special attention has been given to arbuscular mycorrhizal fungi (AMF), concluding that the transformation to agriculture reduces its richness and diversity (Moora et al., 2014; Xiang et al., 2014; Verbruggen et al., 2015). The minority of soil fungal studies addresses communities in drylands (Vargas-Gastélum et al., 2015), only a few of them have targeted the effect of the water quantity and quality used for crop irrigation on the soil fungal community composition, diversity and function in dry regions (Ortega-Larrocea et al., 2001; Alguacil et al., 2012; Disciglio et al., 2015; García-Orenes et al., 2015).

In this study, and for the first time, the soil fungal community composition is compared between semiarid shrubland and agricultural land irrigated with different water qualities. We determined the soil fungal community composition and diversity of natural dryland vegetation (shrubland), rainfed system, and irrigation agriculture with different water qualities during the dry and rainy seasons using amplicon-based MiSeq sequencing of the internal transcribed spacer 2 (ITS2) region. DNA-based analysis to assess total fungal community was employed and FUNGuild (Nguyen et al., 2016) was used to evaluate specific fungal trophic modes. The study was conducted in the area known as Mezquital Valley, which is located in central Mexico and covers 90,000 ha. This region represents the largest continuous area in the world in where crop irrigation is performed with untreated wastewater (Siebe and Cifuentes, 1995; Siemens et al., 2008). The soil of this area provides two important services: (a) filtering the untreated wastewater recharging the aquifer, which is used by the surrounding population for consumption and recreational activities, and (b) crop production, mainly fodder (lucerne, oat, rye grass, as well as maize) (Lüneberg et al., 2018). In 2014 more than 4 million tons of fodder crops and vegetables were produced in this area (Conagua and Semarnat, 2015).

We stressed the hypotheses (i) that land use change and different irrigation practices in the Mezquital Valley area affect fungal diversity and community composition. It has been shown that fungal community structure was affected by land use change in different ecosystems (Orgiazzi et al., 2012; Lupatini et al., 2013; McGuire et al., 2015; Brinkmann et al., 2019). Irrigation practices and seasonality modify soil moisture, especially in drylands, which is an important driver of fungal activity (Tedersoo et al., 2014; Vargas-Gastélum et al., 2015), and thus, we hypothesize that (ii) seasonality plays a role in structuring fungal communities, and soil moisture is the strongest driver, followed by nutrient availability. Due to previous studies reporting an increased frequency of saprophytic and plant pathogenic fungi in irrigated and fertilized soil (Calderon et al., 2016; Zheng et al., 2017), and a decrease of AMF in wastewater irrigated soil (Ortega-Larrocea et al., 2001; Alguacil et al., 2012), we assume that (iii) saprophytic and pathogenic fungi will increase and AMF will decrease in soils under periodic irrigation with wastewater. This study will help to better understand the impact of environmental manipulation on fungal communities in drylands, especially of those taxa involved in essential functional aspects of these soil ecosystems.

## Materials and Methods

### Study Sites and Sample Collection

The sampling area was described by Lüneberg et al. (2018). Briefly, The Mezquital Valley has a semiarid climate with average annual temperatures from 16 to 18°C and an average annual rainfall from 400 to 600 mm (British Geological Survey [BGS], 1998). The natural vegetation in the area is classified as xerophytic shrubs with mesquite (*Prosopis juliflora*) as the dominant tree species. The detailed description of the study area and a map showing the sampling locations can be found in the Supplementary Text S1 and Supplementary Figure S1.

We chose sampling plots with different land use systems and with clay to silty clay loam texture, as it is one of the representative soil types of the area (Haplic Phaeozem; IUSS Working Group WRB, 2014). The sampling encompassed four plots × five land use systems × four samplings. The land use systems included natural dryland vegetation (shrubland plots S1–S4), rainfed plots (R1–R4), plots irrigated with freshwater (FW1–FW4), plots irrigated with wastewater coming from the Endhó dam (DWW1–DWW4) and plots irrigated with untreated wastewater (UTWW1–UTWW4) (Supplementary Figure S1). The size of the plots ranged among one to two ha. The sampling was performed twice during the rainy season (June – October) and twice during the dry season (November – May). All the plots used for agriculture were cropped with maize during the rainy season. During the dry season the rainfed plots were abandoned and the irrigated plots were fallow or cropped with lucerne, oat or grass. At each sampling plot, 20 bulk soil cores from the upper 10 cm were sampled in a regular systematic grid. The cores were homogenized and pooled into one composite sample per plot. The sample was frozen in liquid nitrogen and stored at −80°C until DNA extraction.

### Soil Properties

The soil samples were air-dried for 24 h, homogenized and sieved using a metallic mesh (2 mm). The physical and chemical properties were determined using standard procedures (Soon and Hendershot, 1992). To determine soil pH, 10 g of each soil sample were suspended at a soil-to-liquid ratio of 1:2 (soil – 0.01 M CaCl2). Subsequently, the pH in the supernatant was measured with a glass electrode (Conductronic pH 120, Puebla, Mexico). The gravimetric soil water content (%) was calculated from oven-dried subsamples. Soil organic C content and total N was determined using a Perkin Elmer 2400 CHNS/O elemental analyzer (Massachusetts, United States). Available P was determined by spectrophotometry (Genesis 20, Massachusetts, United States) based on the methodology developed by Olsen (van Reeuwijk, 2002). Soil particle size distribution was determined using the Bouyoucos hydrometer method. The electrical conductivity was determined in a 1:2 soil:distilled water suspension with a conductivity meter (Hanna HL4321, Rhode Island, United States). Exchangeable bases (Ca^2+^, Mg^2+^, Na^+^ and K^+^) were extracted with 1N NH_4_OAc, and the determination of Ca^2+^ and Mg^2+^ was done by atomic absorption spectrophotometry (Perkin Elmer 3110, Massachusetts, United States), for Na^+^ and K^+^ flame emission spectrometry (Sherwood Scientific 36, Cambridge, United Kingdom), according to van Reeuwijk (2002). Table 1 shows the main soil properties. Written and graphic description of soil properties can be found in the Supplementary Text S2 and Supplementary Figure S2.

**Table 1:** Soil properties.

	Shrubland	Rainfed	FW	DWW	UTWW
Soil moisture (%)	18.16 ± 2.2 a	18.24 ± 2.5 a	18.68 ± 2 a	34.5 ± 3.8 b	33.27 ± 2 b
Ph	7.07 ± 0.12 b	7.76 ± 0.06 a	7.79 ± 0.09 a	7.63 ± 0.09 ac	7.32 ± 0.08 bc
E.C. (microS cm^−1^)	1152 ± 172.3 a	973.4 ± 71.2 a	1362 ± 202.3 ab	1337 ± 131.7 ab	1660 ± 89.4 b
C (mg g^−1^)	31.94 ± 4.9 a	14.56 ± 0.9 b	26.66 ± 3.4 a	20.25 ± 1.2 ab	23.43 ± 0.9 a
N (mg g^−1^)	2.8 ± 0.4 a	1.23 ± 0.1 b	1.96 ± 0.2 a	2.04 ± 0.1 a	2.41 ± 0.1 a
C:N	11.54 ± 0.2 a	9.77 ± 0.2 b	12.96 ± 0.7 a	9.93 ± 0.3 b	11.88 ± 0.4 a
P (mg kg^−1^)	27.03 ± 5.5 a	13.05 ± 1.9 a	43.98 ± 14.2 a	54.15 ± 7.2 b	73.86 ± 3.4 b
Ca (g kg^−1^)	16.53 ± 1.4 a	16.3 ± 0.7 a	14.97 ± 1.5 a	15.65 ± 1.4 a	9.94 ± 0.4 b
Mg (g kg^−1^)	1.83 ± 0.2 a	1.63 ± 0.2 a	1.79 ± 0.2 a	2.49 ± 0.1 b	2.76 ± 0.1 b
K (g kg^−1^)	0.57 ± 0.05 a	0.71 ± 0.08 a	1.43 ± 0.3 b	1.08 ± 0.06 b	1.36 ± 0.08 b
Na (g kg^−1^)	0.04 ± 0.01 a	0.08 ± 0.02 a	0.23 ± 0.04 b	0.28 ± 0.03 b	0.49 ± 0.03 c

*Different letters indicate statistical difference (p ≤ 0.05). FW, freshwater irrigated soil; DWW, dam-store wastewater irrigated soil; UTWW, untreated wastewater irrigated soil; E.C, electrical conductivity; P, available Phosphorous; Ca, Calcium; Mg, Magnesium; K, Potassium; Na, Sodium.*

### DNA Extraction and Amplification of ITS2

Soil samples were freeze-dried with liquid nitrogen and kept frozen at −80°C before nucleic acid extraction. Total DNA was extracted by employing the MoBio PowerSoil DNA isolation kit (MoBio Laboratories, Carlsbad, CA, United States) following the manufacturer instructions. DNA concentrations were quantified by using a NanoDrop ND-1000 Spectrophotometer (Thermo Scientific, Schwerte, Germany). Fungal ribosomal internal transcribed spacer (ITS) amplicons were generated using primers ITS3_KYO2 (GATGAAGAACGYAGYRAA) (Toju et al., 2012) and ITS4 (TCCTCCGCTTATTGATATGC) (White et al., 1990) with an Illumina adapter overhang (forward: TCGTCGGCAGCGTCAGATGTGTATAAGAGACAG, reverse: GTCTCGTGGGCTCG GAGATGTGTATAAGAGACAG). For the PCR reaction mixture contained Phusion High Fidelity DNA Polymerase (Thermo Scientific, Schwerte, Germany), 5% DMSO, 0.2 mM of each primer, 1 U DNA Polymerase and 25 ng of isolated DNA as template. Thermal cycling scheme for ITS2 amplicons was as follows: 1 initial min at 98°C, 25 cycles of 45 s at 98°C, 45 s at 48°C, and 30 s at 72°C, and a final extension at 72°C for 5 min. The resulting PCR products were checked by agarose gel electrophoresis for appropriate size and purified by magnetic bead clean-up using the MagSi-NGSPrep Plus as recommended by the manufacturer (MagnaMedics Diagnostics B.V., Geleen, Netherlands). Quantification of the PCR products was performed using the Quant-iT dsDNA HS assay kit and a Qubit fluorometer (Invitrogen GmbH, Karlsruhe, Germany) following the manufacturer’s instructions. All PCR reactions were performed in triplicate and pooled in equal amounts. Pooled PCR products were used to attach indices and Illumina sequencing adapters using the Nextera XT Index kit (Illumina, San Diego, CA, United States). Index PCR was performed using 5 μl of template PCR product, 2.5 μl of each index primer, 12.5 μl of 2x KAPA HiFi HotStart ReadyMix and 2.5 μl PCR grade water. Thermal cycling scheme was as follows: 95°C for 3 min, 8 cycles for 30 s at 95°C, 30 s at 55°C and 30 s at 72°C and a final extension at 72°C for 5 min. Fungal ITS2 region were sequenced using the dual index paired-end 2x 300 bp approach (v3 chemistry) and the Illumina MiSeq platform as recommended by the manufacturer (Illumina).

### Sequence Processing and Analyses

Demultiplexing of raw sequences was performed by CASAVA data analysis software (Illumina). Paired-end sequences were merged using PEAR v0.9.10 (Zhang et al., 2014) with default parameters. The resulting sequences that fulfilled at least one of the following criteria were removed with the *split_libraries_fastq.py* script from QIIME 1.9.1: average quality score lower than 20 and containing unresolved nucleotides (Caporaso et al., 2010). Reverse and forward primer sequences were removed by employing cutadapt v1.12 (Martin, 2011) with default settings. In preparation for the generation of amplicon sequencing variants (ASV), which represents exact sequence variants (Callahan et al., 2017), USEARCH (9.2.64) with the UNOISE2 (Edgar, 2016) algorithm was used to dereplicate (-fastx_uniques), and removed sequences shorter than 140 bp (-sortbylength). After denoising (-unoise2) chimeric sequences were removed using UCHIME2 (Edgar et al., 2011) included in software package USEARCH (9.2.64) in *de novo* and reference mode (-uchime2_ref) against UNITE database (v7.1)^1^ (Koljalg et al., 2013). All quality-filtered sequences were mapped to chimera-free ASVs and an ASV abundance table was created using USEARCH (-usearch_global) with default settings. Taxonomic classification of the picked reference sequences (ASV) was performed with *parallel_assign_taxonomy_blast.py* against the UNITE database (v7.1)^1^ (Koljalg et al., 2013). Extrinsic domain ASVs and unclassified ASV were removed from the data set by employing *filter_otu_table.py*. Finally, all unidentified fungal ASVs were searched with BLAST (blastn) (Altschul et al., 1990) against the nt database (version from March 2017) to remove non-fungal ASVs and only as fungi classified reads were kept. Sample comparisons were performed at the same surveying effort, utilizing the lowest number of sequences by random selection (total 16,800). Species richness, alpha and beta diversity estimates and rarefaction curves were determined using the QIIME 1.9.1 script *alpha_rarefaction.py*.

### Prediction of Fungal Trophic Modes

Fungal trophic modes were predicted from the resulting ASV table using the software package FUNGuild (Nguyen et al., 2016). Fungal ASVs assigned only to one trophic mode (symbiotrophy, saprotrophy, and pathotrophy) were included in the analyses, as environmental conditions can modify the guild of specific groups (Nguyen et al., 2016). For pathothrops the abundance of plant and animal pathogens was analyzed separately. The abundances of symbiotrophs of arbuscular mycorrhizal (AM) and ectomycorrhizal (EcM) fungi were also analyzed separately.

### Identification of Human Pathogens

The rarefied soil and wastewater ASV tables were screened for genera containing potentially human pathogenic members such as *Ajellomyces, Alternaria, Apophysomycetes, Aspergillus, Bipolaris, Blastomyces, Candida, Cephaliophora, Cladosporium, Coccidioides, Colletotrichum, Cryptococcus, Cunninghamella, Curvularia, Fonsecaea, Fusarium, Histoplasma, Lasiodiplodia, Lichtheinia, Malassezia, Microsporum, Mucor, Paracoccidioides, Penicillium, Pneumocystis, Rhizomucor, Rhizopus, Rhodotorula, Scedosporium, Schizophyllum, Sporothrix*, and *Trichophyton* based on the information provided by Mycology Online of the University of Adelaide, Australia^2^. This search was made using QIIME 1.9.1 script *filter_taxa_from_otu_table.py*. The corresponding sequences were double checked against the nucleotide collection of the NCBI using blastn. ASVs that exhibited > 98% identities to known pathogenic members of the genus were further analyzed. The pathogenic nature of the thereby identified taxa was confirmed by literature searches. Taxa for which no pathogenic features were described were discarded.

### Statistical Analyses

All statistical analyses were conducted employing R version 3.3.1 (R Core Team, 2016). The results of all statistical tests were regarded significant with *p* ≤ 0.05. To evaluate the differences among land use systems and between seasons we employed a generalized mix effect model with the land use as a fix effect, followed by Bonferroni’s multiple comparison tests (Shannon index, observed species, and fungal trophic modes). Kruskal-Wallis and Dunn’s tests were used to determined differences among land use systems for the most abundant phyla and potential human pathogens. To evaluate the correlation of diversity indexes and fungal trophic modes with soil properties Spearman’s rank correlation test was employed. To identify the fungal families associated with the different land use systems, an analysis based on the point biserial correlation coefficient was performed using *multipatt* (package “indicSpecies”) (de Cáceres, 2013). For visualization, a network was generated using the land use systems as source nodes, and the fungal families as target nodes. All taxa with significant (*p* ≤ 0.05) associations were visualized in the networks. Network generation was performed using the *edge-weighted spring embedded layout* algorithm in *Cytoscape* (Shannon et al., 2003), with the edge weight corresponding to the association weight of each family with each land use system. To evaluate and graph the most abundant genera we employ ampvis2 (Andersen et al., 2018), unidentified ASVs were removed, color scale of abundance was square rooted to better visualize low abundant taxa. To assess the (dis)similarity of fungal communities between land use systems and season ANOSIM test was performed in QIIME (Caporaso et al., 2010) using Bray-Curtis dissimilarities with the script *compare_categories.py*. To visualize the multivariate dispersion of community composition a non-metric multidimensional scaling (NMDS) was performed using Bray-Curtis dissimilarities employing the “vegan” package (Oksanen et al., 2015). Standard deviation ellipses by land use system were projected onto the ordination, utilizing the function *ordiellipse*. The effects of environmental parameters on the fungal community were analyzed using the *envfit* function and significant correlated parameters were projected into the ordination with arrows. In order to evaluate the multicollinearity among environmental parameters, a Spearman’s rank correlation test was performed and the variance inflation factors (VIF) were determined (Supplementary Table S1). As nitrogen and carbon were highly correlated (*R*^2^ > 7 and the VIF value of nitrogen was > 4), nitrogen was removed from the analysis.

### Sequence Data Deposition

The ITS2 region sequences were deposited in the National Centre for Biotechnology Information (NCBI) Sequence Read Archive (SRA) under bioproject accession number PRJNA386070.

## Results

### General Characteristics of the ITS Dataset

To analyze the fungal community structure, DNA was isolated from 80 soil samples derived from all analyzed land use systems. Despite several attempts with modified conditions, the isolation of high quality DNA from one rainfed sample (R1D1) failed. After removal of low-quality sequences and singletons, amplicon-based analysis of the ITS2 region of the fungal rRNA resulted in 5,645,373 high-quality sequences. The number of sequences per sample ranged from 16,830 to 342,599. After rarefaction analysis with the minimal amount of sequences per sample (16,800), 9,685 ASVs were obtained (773 + 203 per sample) (Supplementary Figure S3). The Good’s coverage index of 0.98 (+ 0.004) indicates that the dataset enclose all major fungal groups inhabiting the studied land use systems.

### ASVs Richness and Diversity

**FIGURE 1:**
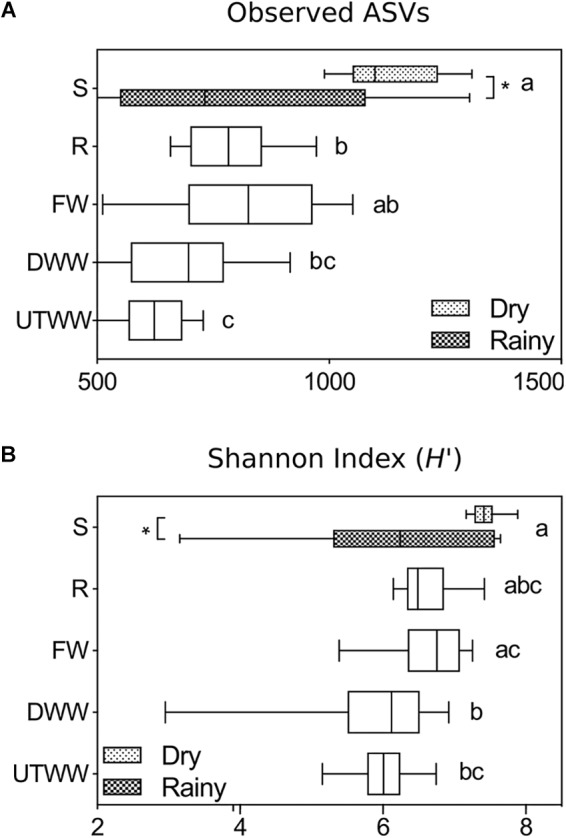
**(A)** Richness and **(B)** diversity indices of the total fungal communities in Shrubland (S), Rainfed (R), Freshwater (FW), Dam wastewater (DWW), and Untreated wastewater (UTWW) irrigation systems, during the dry and rainy season. Box are extended from the 25th to 75th percentiles, the line in the box is plotted at the median. Whiskers represent the smallest and the largest value. A mix effect model followed by Bonferronis multiple comparison tests were used to determine differences among land use systems, and between seasons. Different letters indicate statistical difference among land use systems and ^∗^ indicate statistical differences between seasons (*p* ≤ 0.05). Only parameters differing significantly between seasons in each land use system are shown, if they did not differ, samples of both seasons were merged.

Fungal diversity (Shannon index *H′*) and ASVs richness (number of observed ASVs) responded to land use change and water used for irrigation (*p* ≤ 0.05, Figure 1). The diversity of the fungal community was largest in the shrubland soil (*H′* 6.8), mainly during the dry season (*H′ 7.4*) and smallest in the wastewater-irrigated fields (*H′* 6.0). The same was found for the ASVs richness, which were higher in shrubland soil, in average 956 ± 72, compared to 619 ± 18 from the UTWW system. The shrubland ASV richness was especially higher during the dry season 1123 ± 39 compared to 789 ± 112 found during the rainy season (*p* ≤ 0.05).

The evaluation of soil properties impact on the fungal diversity and ASVs richness (Table 2) showed a strong negative correlation to soil moisture and Na^+^ content, and a weaker but significant negative correlation to available P, K^+^, and Mg^2+^ content (*p* ≤ 0.05).

**Table 2:** Spearman correlation coefficient between diversity metrics and soil properties.

	Moisture (%)	pH	C.E. (μS cm^−1^)	C (mg g^−1^)	N (mg g^−1^)	C:N	P (mg kg^−1^)	Ca (g kg^−1^)	Mg (g kg^−1^)	K (g kg^−1^)	Na (g kg^−1^)
Observed ASVs	**−0.54**	−0.17	−0.12	−0.08	−0.04	0.12	**−0.42**	0.15	**−0.30**	**−0.40**	**−0.54**
Shannon	**−0.59**	−0.1	−0.08	−0.09	−0.05	**0.25**	**−0.37**	0.15	**−0.31**	**−0.30**	**−0.53**

*Bold numbers indicate significance (p ≤ 0.05). E.C, electrical conductivity; P, available Phosphorous; Ca, Calcium; Mg, Magnesium; K, Potassium; Na, Sodium.*

### Fungal Community Composition

**FIGURE 2:**
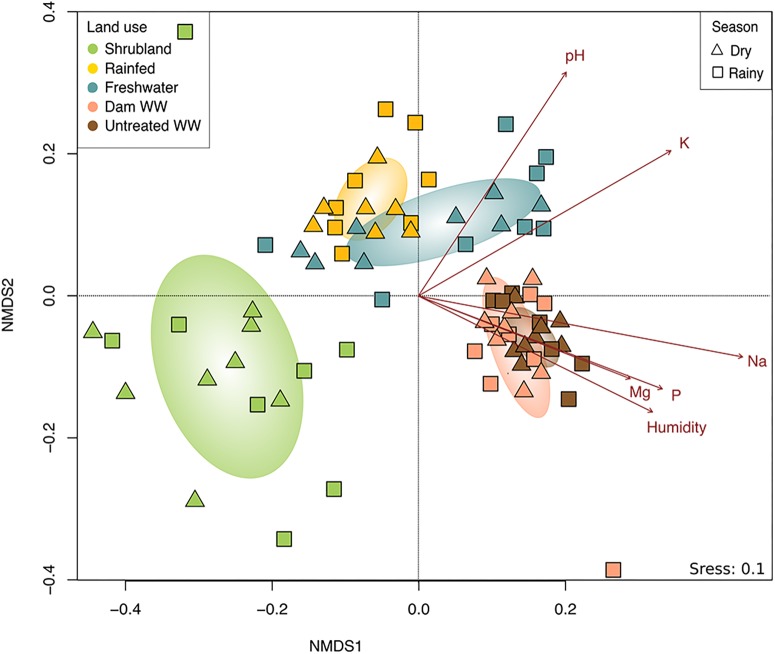
Non-metric multidimensional scaling (NMDS) of the total fungal community composition from land use systems, shrubland, rainfed, freshwater, dam wastewater and untreated wastewater irrigation and dry and rainy season. Soil parameters with strong and significantly correlation (*p* ≤ 0.05) to the fungal community structure are indicated by arrows.

The composition of fungal communities differed among land use systems as indicated by ANOSIM test (*p* = 0.001). The (dis)similarities seem to be related to whether the land was untilled or used for agriculture. Shrubland samples were separated from the other land use systems, while the communities of rainfed and FW showed certain similarity (Figure 2), and the communities irrigated with wastewater (DWW and UTWW) were highly akin. Based on the ANOSIM test, none of the studied land use systems differed seasonally, however, during the dry season, the agricultural fields were either abandoned, fallow or cropped with oat, grass or lucerne.

To evaluate for multicollinearity of the soil physico-chemical properties a Spearman correlation test was performed and the VIF was determined. Total C and N showed high correlation (*R*^2^ > 7) and N obtained a VIF > 4. Based on these results we removed N from the analysis. After fitting the soil parameters onto the ordination, we found that exchangeable sodium content exhibited the strongest effect on the fungal community (*R*^2^ = 0.62, *p* = 0.001), followed by soil pH and moisture (*R*^2^ = 0.55, *R*^2^ = 0.37, respectively, *p* = 0.001; Figure 2). Interestingly, soil base cations (Mg^2+^, K^+^, and specially Na^+^) played an important role in the composition of the fungal community, being almost as important as soil humidity, and even more important than the nutritional status of soil (P content and C:N ratio).

### Taxonomic Groups

**FIGURE 3:**
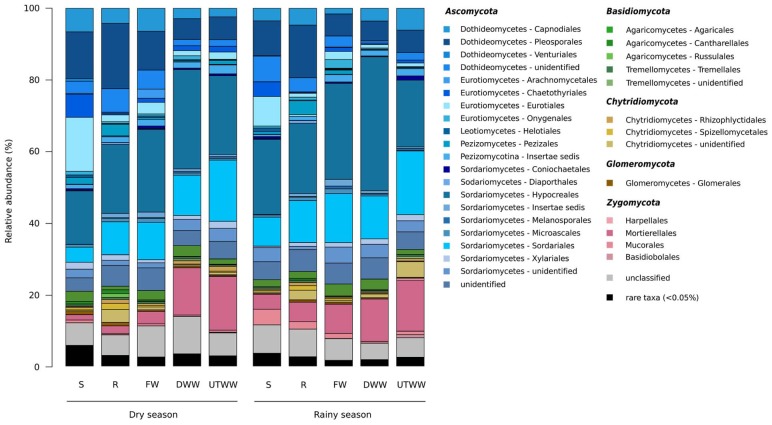
Relative abundances of soil fungal orders. Land use systems: Shrubland (S), Rainfed (R), Freshwater (FW), Dam wastewater (DWW) and Untreated wastewater (UTWW) irrigated, during dry and rainy season. Bacterial orders with average relative abundances >0.5% is visualized; orders contributing ≤0.5% were summarized as rare taxa.

**FIGURE 4:**
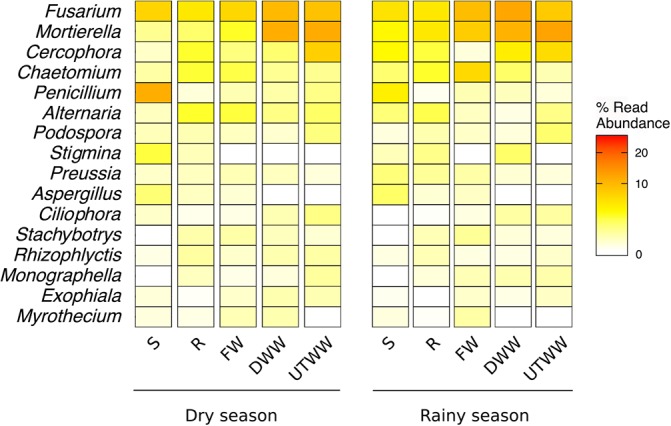
Top 20 most abundant fungal genera. Land use systems: Shrubland (S), Rainfed (R), Freshwater (FW), Dam wastewater (DWW), and Untreated wastewater (UTWW) irrigated, during dry and rainy season.

The ASVs across all samples were assigned to five fungal phyla and more than 54 orders. The studied land use systems were dominated by *Ascomycota* (75.4%), followed by *Zygomycota* (9.4%), *Basidiomycota* (4.4%), *Chytridiomycota* (3.1%), and *Glomeromycota* (0.6%) (Figure 3). At order level, *Hypocreales* (23%), *Sordariales* (11.5%), *Pleosporales* (9.7%), and *Mortierellaes* (7.9%) were predominant. The most abundant identified genera were *Fusarium, Mortierella, Cercophora, Chaetomium*, and *Penicillum* (Figure 4). The relative abundance of *Ascomycota* diminished in the UTWW system (*p* ≤ 0.05), while *Zygomycota* and *Rozellomycota* increased under wastewater irrigation (*p* ≤ 0.05). The other phyla did not show significant abundance changes among land use systems (Supplementary Figure S4).

**FIGURE 5:**
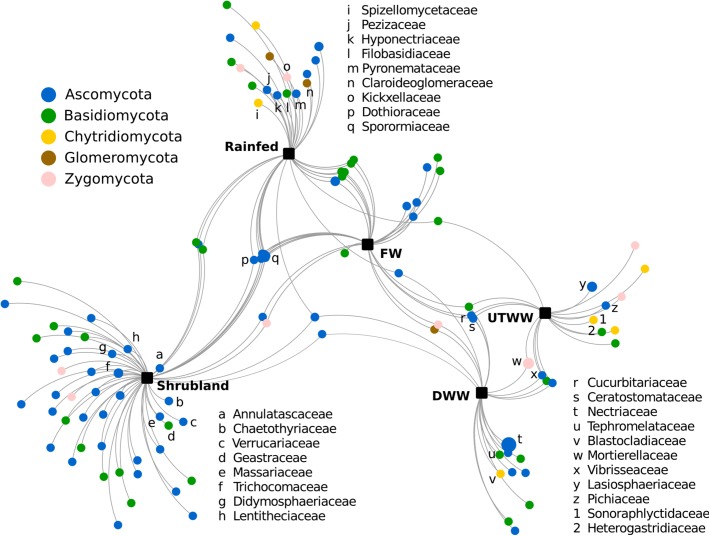
Correlation-based association network at family level of the fungal communities among land use systems: shrubland, rainfed, freshwater, irrigated with dam and untreated wastewater, respectively. Only statistical significant fungal families are visualized (*p* ≤ 0.05). The size of the nodes is proportional to the taxon relative abundance and the edge width corresponds to the association strength of each taxon with the land use system.

To identify families significantly associated with one, two, or more land use systems, a correlation-based association analysis was performed. Here, all fungal families were included, however, on average the relative abundance of 52% of fungal families were not significantly different with respect to the land use system. The correlation-based association analysis (Figure 5) was consistent with the multivariate analysis (Figure 2). The fungal community from shrubland soil was separated from the agricultural land use systems, representing the system with the highest number of associated taxa.

Most fungal taxa significantly associated to shrubland soil belong to *Ascomycota*, the families with stronger association were *Annulatascaceae, Chaetothyriaceaea, Verrucariaceae, Geastraceae, Massariaceae, Trichomaceae, Didymosphaeriaceae, Lentitheciaceae, Teloschistaceae*, and *Parmeliaceae* (Figure 5). The latter was also associated to rainfed soil, also associated to rainfed soil were *Spizellomycetaceae, Pezizaceae, Hyponectriaceae, Filobasidiaceae, Pyronemataceae, Claroideoglomeraceae*, and *Kickxellaceae*. Families associated to the three land use systems free from wastewater (shrubland, rainfed, and FW) were *Dothioraceae* and *Sporomiaceae*. The relative abundance of fungal families associated to shrubland, rainfed and partially FW had a negative correlation to soil moisture, Na^+^ content, pH, and available P (Supplementary Table S2). Some of these taxa are *Annulatascaceae, Geastraceae, Massariaceae, Trichocomaceae, Didymosphaeriaceae*, and *Lentitheciaceae*. Most of the taxa associated to rainfed soil were additionally negatively correlated to N content, including, *Hyponectriaceae, Spizellomycetaceae, Filobasidiaceae, Claroideoglomeraceae*, and *Kickxellaceae*. Two of the families associated to the land use systems under periodic irrigation (FW, DWW, and UTWW) were *Cucurbitariaceae* and *Ceratostomataceae*. Among the families that were significantly associated to systems under wastewater irrigation we detected *Nectriaceae, Tephromelataceae, Blastocladiaceae, Mortierellaceae, Vibrisseaceae, Pichiaceae, Lasiosphaeriaceae, Heterogastridiaceae*, and *Sonoraphlyctidaceae* (Figure 5). Most families associated to the wastewater irrigated systems were in contrary to shrubland and rainfed systems positively correlated to soil moisture, Na^+^ content and available P (Supplementary Table S2).

### Fungal Trophic Modes

**FIGURE 6:**
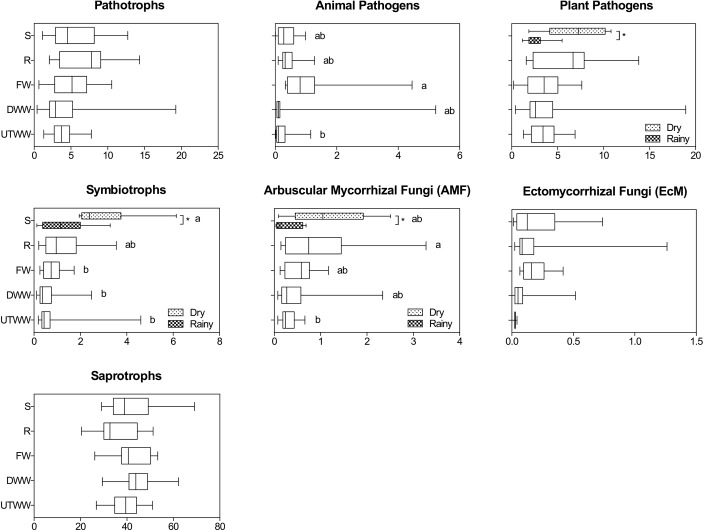
Relative abundances of fungal trophic modes within each land use system. Shrubland (S), Rainfed (R), Freshwater (FW), Dam wastewater (DWW), and Untreated wastewater (UTWW) during the dry and rainy season. Boxes extend from the 25th to 75th percentile, the line in the box is plotted at the median. Whiskers represent the smallest and the largest value. A mix effect model followed by Bonferroni’s multiple comparison tests were used to determine differences among land use systems, and between seasons. Different letters indicate statistical difference among land use systems and ^∗^ indicate statistical differences between seasons (*p* ≤ 0.05). Only parameters differing significantly between seasons in each land use system are shown, if they did not differ, samples of both seasons were merged.

Saprotrophy was the predominant trophic mode in all land use systems, showing no significant differences in relative abundances (Figure 6). The most abundant saprotrophs belonged to the *Nectriaceae, Lasiosphaeriaceae*, and *Mortierellaceae* families. The 20 most abundant saprothrophic genera are depicted in the Supplementary Figure S5. The abundance of saprotrophs was positively correlated to total C and available P in the soil, *R*^2^ = 0.25 and 0.32, respectively (*p* ≤ 0.05; Table 3). Symbiotrophic fungi showed a decrease in the land use systems under periodic irrigation (FW, DWW, UTWW) in comparison to shrubland and rainfed systems (*p* ≤ 0.05). AMF had a higher relative abundance in the rainfed soil and lower in the UTWW system. The relative abundance of symbionts and AMF was higher during the dry season in the shrubland soil. The most abundant symbiothrophic genera were *Capronia*, which was only detected in the Shrubland soil and, *Glomus* that was detected in the land use systems under periodic irrigation (Supplementary Figure S6). The relative abundance of EcMF did not show differences among land use systems. The most abundant AMF genus was *Glomus* and the most abundant EcMF genera were *Pezisa* and *Amanita.* Symbiotrophs in general were negatively associated to humidity and available P (*R*^2^ = −0.30). EcMF were also negatively associated to base saturation cations, which are increased in wastewater irrigated systems (*p* ≤ 0.05; Table 3). The abundance of pathotrophs including plant and animal pathogens was not higher in the wastewater-irrigated systems than in the other systems, the same was observed specifically for plant pathogens. The relative abundance of animal pathogens was higher in the FW system and lower in the UTWW one. During the dry season the abundance of plant pathogens was higher in the shrubland soil. Three of the most abundant plant pathogens detected were *Bipolaris cynodontis, Lectera colletotrichoides*, and *Gibellulopsis nigrescens* (Supplementary Figure S7). The most abundant animal pathogenic taxa detected were *Metarhizium marquandii*, *M. anisopliae*, and *Metacordyceps chlamydospori.* Pathotrophs in general showed negative correlation to several physico-chemical soil properties enhanced in wastewater irrigation systems, such as humidity, E.C., total N, K^+^, and Na^+^ content and available P (*p* ≤ 0.05; Table 3).

**Table 3:** Spearman correlation coefficient between fungal trophic modes and soil properties.

	Moisture (%)	pH	C.E. (μS cm^−1^)	C (mg g^−1^)	N (mg g^−1^)	C:N	P (mg kg^−1^)	Ca (g kg^−1^)	Mg (g kg^−1^)	K (g kg^−1^)	Na (g kg^−1^)
Saprotrophs	0.079	0.045	0.014	**0.256**	0.199	0.135	**0.329**	0.022	−0.055	0.179	−0.039
Symbionts	**−0.303**	−0.135	−0.033	0.043	0.026	0.18	**−0.302**	0.15	−0.199	**−0.29**	**−0.354**
EcMF	**−0.402**	0.161	−0.163	−0.135	−0.221	**0.385**	**−0.495**	**0.279**	**−0.351**	**−0.334**	**−0.372**
AMF	**−0.285**	0.084	0.047	−0.135	−0.173	0.164	**−0.299**	0.131	−0.123	−0.161	−0.129
Pathotrophs	**−0.326**	−0.107	**−0.297**	−0.2	**−0.328**	0.117	**−0.326**	0.022	−0.1	**−0.357**	**−0.235**
Plants	−0.219	−0.165	−0.183	−0.151	**−0.247**	0.059	**−0.223**	0.058	0.034	**−0.27**	−0.094
Animals	**−0.334**	0.167	0.003	0.046	−0.121	**0.377**	**−0.312**	0.137	−0.205	−0.088	−0.196

*Bold numbers indicate significance (p ≤ 0.05). EcMF, Ectomycorrhizal fungi; AMF, Arbuscular mycorrhizal fungi; E.C, electrical conductivity; P, available Phosphorous; Ca, Calcium; Mg, Magnesium; K, Potassium; Na, Sodium.*

To determine whether the wastewater increased the abundance of human pathogens in soil we searched for specific genera known for their pathogenicity in soil and wastewater samples. Pathogenic species of *Aspergillus, Candida, Fusarium, Malassezia*, and others were found in the wastewater samples (Supplementary Table S3). The shrubland system had a lower abundance of human pathogens, while higher abundances were detected in the wastewater-irrigated systems than in the rainfed and FW systems. However, this difference was not statistically significant. In the wastewater-irrigated systems the most abundant species were *Bipolaris cynodontis, Candida quercitrusa*, and *Fusarium fujikuroi* (*p* ≤ 0.05) (Supplementary Figure S8).

## Discussion

In this study we evaluated the effect of changing soil physical and chemical parameters due to land use change and the quality of water used for irrigation on the diversity and composition as well as the functionality of fungal community from a dryland soil. The study was conducted in the Mezquital Valley, which provides representative conditions to study soil fungal communities under different land use systems, including preserved natural vegetation (shrubland), rainfed agriculture or irrigated agriculture with three different water qualities: freshwater, untreated wastewater stored in a dam and untreated wastewater.

### Irrigation With Wastewater Decreases Fungal Richness and Diversity

Fungal diversity and richness showed a progressive decrease in the land use systems where irrigation is applied and the quality of water is worsened. The decline of fungal richness and diversity in the DWW and UTWW systems could be related specifically to the higher amount of P that the wastewater introduces into the soil (Siebe, 1998). Shrestha et al. (2015) reported a reduction of fungal diversity in fertilized soils in comparison to non-amended soil. The moisture effect in the fungal diversity and richness is also evident as the systems under wastewater irrigation were more humid than shrubland, rainfed and FW. These results are in line with several previous studies reporting a more abundant and diverse fungal community under drought or dry soil conditions, and less abundant and diverse emerging during wetter periods and in wetter soils (Hawkes et al., 2011; Acosta-Martínez et al., 2014; Ma et al., 2015; Zheng et al., 2017). The thriving of fungi in dryer conditions is related to the capability of fungal hyphae to grow in small pores and take up water that may be retained in them, as they do not experience the limitation of movement that is inherent to a unicellular structure (Chowdhury et al., 2011; Mchugh et al., 2014). Shrubland soil was the only system that showed differences between seasons, probably several groups encompassing this community are more sensitive to changes in water content that are not present in the other systems. Interestingly, fungal diversity and richness was also negatively correlated to the content of exchangeable soil cations Na^+^, K^+^, and Mg^2+^, which are enhanced in wastewater-irrigated soil. Specifically, the effect of Na^+^ content and soil salinity on fungi is still unclear (Rath and Rousk, 2015). Some studies (Chowdhury et al., 2011; Baumann and Marschner, 2013) have reported a decreased proportion of fungal phospholipid fatty acids (PLFAs) with higher salinity. Sardinha et al. (2003) reported a decrease in the fungal biomarker ergosterol with increasing salinity under acidic soil conditions. Also, salt affected soils have shown a lower fungal to bacteria ratio (Pankhurst et al., 2001). Salinity can also have a negative effect on AM fungi, however, several reports showed improved growth and performance of mycorrhizal plants under salt stress conditions (Porcel et al., 2012). It cannot be excluded that contaminants present in the wastewater not measured in this study have affected the diversity of the fungal community.

In the soil of the Mezquital Valley, bacteria responded differently than fungi. Bacteria had higher diversity in the agricultural system under freshwater irrigation than in the shrubland system, bacterial diversity was correlated positively to pH and Ca^2+^ and negatively to P and Na^+^ content (Lüneberg et al., 2018), this suggests that the soil conditions caused by wastewater irrigation affect the diversity of fungi and bacteria, but fungi are affected more severely.

### Fungal Community Composition Is Affected by Land Use

The soil fungal community composition of the Mezquital Valley is influenced primarily by whether the land conserved its natural vegetation or if it was use for agriculture, and secondly by the quality of water use for irrigation. The clustering according to land use system shows the importance of interactions between fungal community structure and vegetation diversity, as shrubland fungal community is separated from the monocultural land use systems. Similar trends were recorded during conversion of temperate and tropical forests into plantation (Kasel et al., 2008; Purahong et al., 2014; McGuire et al., 2015) or grassland (Lauber et al., 2008; Lopes Leal et al., 2013), and when natural grassland was transformed to agricultural systems (Bissett et al., 2011; Xiang et al., 2014). Regarding agricultural management, many studies focused on practices such as fertilization, tillage, crop rotation and organic farming (Morris et al., 2013; Moora et al., 2014; Wang et al., 2016), but not on water quality used for irrigation. Here, soil fungal structure shifts radically in soil under wastewater irrigation, similar to the findings of Disciglio et al. (2015) for treated wastewater irrigated soil.

The strong impact of Na^+^ content, as well as K^+^ and Mg^2+^ on the composition of the fungal community agrees with reports in agricultural land and Mediterranean and semiarid sites, where base saturation was reported to alter total fungal or AMF community structure (Jansa et al., 2014; Ramalho da Silva et al., 2014; Ciccolini et al., 2015). Additionally, in a dry forest Gonçalves Lisboa et al. (2014) found that fungi to bacteria ratio was related to soil cations. The effect of base saturation can be related to the inhibition of spore germination, hyphal development and the production of arbuscules by salinity, as has been observed for AMF (Miransari, 2010). Compared to the well-established role of pH in the determination of soil bacterial composition, the effect of pH on the soil fungal composition is still under debate. On one hand, studies reported pH as one of the main drivers of total and AM fungal composition (Rousk et al., 2010; Jansa et al., 2014; Moora et al., 2014; Qin et al., 2015), or a pH effect on the abundance of fungi (Tedersoo et al., 2014; Maestre et al., 2015) and the fungi to bacteria ratio (Rousk et al., 2010). On the other hand, studies are available that did not identify soil pH as a driver of the fungal community composition (Lauber et al., 2008; Ramalho da Silva et al., 2014; Ciccolini et al., 2015). Here, pH was one of the strongest drivers of the community given the narrow pH range of the soils (7.1–7.8). In general, fungi show optimal growth under a wide pH range (5–9), although different taxa of soil fungi showed preference for certain pH values (Rousk et al., 2009). However, pH is directly or indirectly related to different soil properties such as nutrient availability, solubility of metals and salinity (Lauber et al., 2009; Rousk et al., 2009).

The composition of bacteria in the Mezquital Valley soils was also determined by Na^+^ content, pH and soil moisture (Lüneberg et al., 2018), which exemplifies the importance of these soil properties when drylands are transformed to agricultural land and are irrigated with untreated wastewater. In the soil of the Mezquital Valley fungi seem to be more sensitive to salinity than bacteria, this is in line with the report of Baumann and Marschner (2013).

The fungal community of this study was dominated by *Ascomycota* and *Basidiomycota* as previously reported for most dryland and agricultural soils (Steven et al., 2014; Maestre et al., 2015; Wang et al., 2016). The structural shifts in fungal communities with a decrease of *Ascomycota* and the increase of *Zygomycota* and *Rozellomycota* in the wastewater-irrigated systems indicate lineage-specific adaptations of higher taxonomic groups. The correlation-based analysis of associated taxa showed half of the groups were associated to one or more land use systems. Most fungal taxa significantly associated to shrubland soil belong to the classes *Eurotiomycetes* and *Dothideomycetes* of the *Ascomycota* phylum; this agrees with previous reports that have shown *Ascomycota* dominating fungal communities of semiarid grasslands and drylands (Porras-Alfaro and Bayman, 2011; Maestre et al., 2015; Vargas-Gastélum et al., 2015). Specifically *Eurotiomycetes* have been associated to abandoned agricultural soil compared to maize monoculture system (Ciccolini et al., 2015). Among the taxa associated to shrubland, several lichenized families were identified, such as *Chaetothyriaceae, Verrucariaceae, Teloschistaceae*, and *Parmeliaceae*, the two latter belong to the class *Lecanoromycetes*, from where are the most lichen fungi that form symbioses with filamentous *Nostoc* cyanobacteria (Grube and Wedin, 2016). *Cyanobacteria* was one of the indicator bacterial taxa of shrubland soil (Lüneberg et al., 2018), as well as the order *Rhizobiales* which is a lichen-associated group (Grube and Wedin, 2016). Most of the taxa associated to shrubland and rainfed soil were adapted to drought, hence sensitive to Na^+^ content and P available. Fungal families associated to rainfed soil included two lichen families of the order *Pezizales*. While the taxa associated to wastewater-irrigated soil, such as *Nectriaceae, Tephromelataceae, Blastocladiaceae, Mortierellaceae, Vibrisseaceae, Pichiaceae, Heterogastridiaceae*, and *Trichosporonaceae* were adapted to higher soil moisture, P content and concentration of Na^+^ provided by wastewater irrigation (Siebe, 1998). *Nectriaceae* includes numerous important human and plant pathogens being maize one of its hosts, also several species are used in industrial applications as biodegraders and biocontrol agents (Lombard et al., 2015). The families associated to wastewater-irrigated soil might be also adapted to surfactants and heavy metals present in wastewater (Friedel et al., 2000) not measured here.

### Soil Moisture and Available P Define Fungal Trophic Modes

To translate fungal sequence information into ecologically meaningful information we employed FUNguild (Nguyen et al., 2016) and assigned ASV sequences to trophic modes. The lack of differences in the relative abundance of saprothrophs observed in this study can be related to the wider range of heat and drought conditions, saprophytic populations are able to resist (Acosta-Martínez et al., 2014), this trend was previously observed comparing forest and grassland soil (Nguyen et al., 2016). Our results about the saprotrophic fungi abundance differs from the findings of Disciglio et al. (2015), who reported higher colonization of saprophytic species in soil irrigated with treated wastewater compared to groundwater-irrigated soil. The positive correlation of saprotrophic fungi to total C and N supports the importance of saprothrophs as key regulators of nutrient cycling (Crowther et al., 2012). The abundance of symbiothrophs is related to soil moisture, AMF are known to resist a wide range of heat and drought conditions (Acosta-Martínez et al., 2014), and especially AMF seem to be sensitive to water quality. Previous studies showed specifically a reduced diversity of AMF in treated wastewater irrigated soil due to increase in nutrient availability (Alguacil et al., 2012; Bastida et al., 2017). Additionally, other studies have shown that AM fungal richness and diversity are highly sensitive to available P (Bai et al., 2013; Yang et al., 2014; He et al., 2016). The results we obtained agreed with the previous culture-based study of AMF in the soils of the Mezquital Valley by Ortega-Larrocea et al. (2001), who found a reduction of AMF abundance in soil irrigated with wastewater, and *Glomus* to be the most abundant fungal genus. It is important to notice that the general fungal ITS2 primers used in this study might underrepresent AMF, however, the primers are adequate to evaluate treatment responses (Lekberg et al., 2018). Ectomycorrhizal fungi are primarily associated to trees, its propagation is potentially ceased in agricultural systems, due to irrigation and the use of fertilizers and tillage (Munyanziza et al., 1997; Tedersoo and Smith, 2013; Zheng et al., 2017). The abundance of pathothrophs was not higher in the wastewater irrigated systems as we hypothesized (iii). These results can be possibly related to the higher abundance of bacterial taxa such as *Pseudomonas* in the soil irrigated with wastewater (Lüneberg et al., 2018). *Pseudomonas* are known for their antifungal compound production (Ligon et al., 2000). A similar trend was reported for tomato crops irrigated with secondary-treated agro-industrial wastewater, revealing a decrease of the pathogen *Fusarium* spp. in the soil close to the roots (Disciglio et al., 2015). The most abundant plant pathogens *Bipolaris cynodontis, Lectera colletotrichoides* and *Gibellulopsis nigrescens* are known for infections in maize and lucerne, the main crops of the area (Hu et al., 2011; Inderbitzin et al., 2011; Cannon et al., 2012; Manamgoda et al., 2014). The most abundant animal pathogen taxa detected were *Metarhizium marquandii*, *M. anisopliae*, and *Metacordyceps chlamydospori* which are recognized entomopathogens and pathogens of nematodes and snails (Bischoff et al., 2009; Kepler et al., 2012; Stirling, 2014; Garrido-Jurado et al., 2015; Sanjaya et al., 2016). Surprisingly, most of the taxa associated with distinct land use systems were either negatively or positively correlated with available P. Additionally, P content was the only soil property correlated with the abundance of all the different fungal trophic modes. These results demonstrate the importance of soil nutrient status for the fungal community in each land use system (He et al., 2016). The human pathogens found in the wastewater irrigated systems (*Bipolaris cynodontis, Candida quercitrusa*, and *Fusarium fujikuroi)* produce opportunistic infections in immunocompromised patients (Guarro, 2013; Manamgoda et al., 2014; Xiao et al., 2014). As we could detect more pathogenic species in the systems where wastewater is used for irrigation of the crops, we assume that most pathogenic fungi introduced by wastewater were unable to tolerate the soil conditions.

## Conclusion

In line with the general hypothesis (i) dryland transformation to agricultural land under irrigation with different water quality has a significant effect on the soil fungal diversity and composition. Soil under natural semi-arid shrubland has the highest fungal richness and diversity, while periodic wastewater irrigation negatively affects these fungal indices, by the increment of soil moisture, available P and base saturation content. In accordance with our hypothesis, soil moisture in addition with sodium content and soil pH were the strongest drivers of the fungal community structure. The (dis)-similarities in the structure of the fungal community in the soils of the Mezquital Valley are primarily due to whether the land conserved its natural vegetation or if it was used for agriculture, and secondly by the quality of water used for irrigation. In contrast with our hypothesis (ii), the structure of the fungal community did not differ significantly along the seasons in any of the studied land use systems, despite differences in soil moisture and vegetation cover. The observation from the fungal trophic modes oppose our hypothesis on an increase of saprophytic and pathogenic fungi in soils under periodic irrigation with wastewater (iii); interestingly we observed the opposite, saprotrophic and pathotrophic fungi (including plant and animal pathogens) showed no differences among land use systems. However, three of the most abundant plant pathogens identified are known for infections in maize and lucerne, the main crops of the area. Symbiotic fungi were affected by irrigation and AMF specifically by irrigation with untreated wastewater. The results indicate lineage-specific adaptations to soil properties, as wastewater irrigation reduces the abundance of *Ascomycota*, while the abundance of *Zygomycota* and *Rozellomycota* is increased. We found that fungal families associated to shrubland, rainfed and partially FW were adapted to drought, hence sensitive to exchangeable sodium content and most of them to total N and available P. Taxa associated to freshwater, dam wastewater and untreated wastewater irrigated systems showed the opposite trend. Additionally, the identified potentially harmful human pathogens might be a health risk for the population. The present study contributes to better understand the impact of shrubland transformation into agricultural systems irrigated with different water quality on soil fungal communities in managed semiarid ecosystems.

## Author Contributions

CS, RD, and KL conceived the study. KL carried out the field and laboratory work. DS and KL prepared and analyzed the data. CS, RD, DS, NB, and KL interpreted the results and wrote the manuscript.

## Conflict of Interest Statement

The authors declare that the research was conducted in the absence of any commercial or financial relationships that could be construed as a potential conflict of interest.

## Footnotes

1 https://unite.ut.ee/repository.php

2 https://mycology.adelaide.edu.au/descriptions/

## References

[B1] Acosta-Martínez V. Cotton J. Gardner T. Moore-Kucera J. Zak J. Wester D. (2014). Predominant bacterial and fungal assemblages in agricultural soils during a record drought/heat wave and linkages to enzyme activities of biogeochemical cycling. *Appl. Soil Ecol.* 84 69–82. 10.1016/j.apsoil.2014.06.005

[B2] Alguacil M. M. Torrecillas E. Torres P. García-Orenes F. Roldan A. (2012). Long-term effects of irrigation with waste water on soil AM fungi diversity and microbial activities: the implications for agro-ecosystem resilience. *PLoS One* 7:e47680. 10.1371/journal.pone.0047680 23094075 PMC3475709

[B3] Altschul S. F. Gish W. Miller W. Myers E. W. Lipman D. J. (1990). Basic local alignment search tool. *J. Mol. Biol.* 215 403–410. 10.1006/jmbi.1990.9999 2231712

[B4] Andersen K. S. Kirkegaard R. H. Karst S. M. Albertsen M. (2018). ampvis2: an R package to analyse and visualise 16S rRNA amplicon data. *bioRxiv*

[B5] Bai G. Bao Y. Du G. Qi Y. (2013). Arbuscular mycorrhizal fungi associated with vegetation and soil parameters under rest grazing management in a desert steppe ecosystem. *Mycorrhiza* 23 289–301. 10.1007/s00572-012-0468-5 23179900

[B6] Bastida F. Torres I. F. Romero-Trigueros C. Baldrian P. Vetrovsky T. Bayona J. M. (2017). Combined effects of reduced irrigation and water quality on the soil microbial community of a citrus orchard under semi-arid conditions. *Soil Biol. Biochem.* 104 226–237. 10.1016/j.soilbio.2016.10.024

[B7] Baumann K. Marschner P. (2013). Effects of salinity on microbial tolerance to drying and rewetting. *Biogeochemistry* 112 71–80. 10.1007/s10533-011-9672-1

[B8] Becerra-Castro C. Lopes A. R. Vaz-Moreira I. Silva E. F. Manaia C. M. Nunes O. C. (2015). Wastewater reuse in irrigation: a microbiological perspective on implications in soil fertility and human and environmental health. *Environ. Int.* 75 117–135. 10.1016/j.envint.2014.11.001 25461421

[B9] Bennett J. A. Maherali H. Reinhart K. O. Lekberg Y. Hart M. M. Klironomos J. (2017). Plant-soil feedbacks and mycorrhizal type influence temperate forest population dynamics. *Science* 355 181–184. 10.1126/science.aai8212 28082590

[B10] Birkhofer K. Schöning I. Alt F. Herold N. Klarner B. Maraun M. (2012). General relationships between abiotic soil properties and soil biota across spatial scales and different land-use types. *PLoS One* 7:e43292. 10.1371/journal.pone.0043292 22937029 PMC3425568

[B11] Bischoff J. F. Rehner S. A. Humber R. A. (2009). A multilocus phylogeny of the *Metarhizium anisopliae* lineage. *Mycologia* 101 512–530. 10.3852/07-202 19623931

[B12] Bissett A. Richardson A. E. Baker G. Thrall P. H. (2011). Long-term land use effects on soil microbial community structure and function. *Appl. Soil Ecol.* 51 66–78. 10.1016/j.apsoil.2011.08.010

[B13] Brinkmann N. Schneider D. Sahner J. Ball J. Edy N. Barus H. (2019). Intensive tropical land use massively shifts soil fungal communities. *Sci. Rep.* 9:3403. 10.1038/s41598-019-39829-4 30833601 PMC6399230

[B14] British Geological Survey [BGS] (1998). *Impact of Wastewater Reuse on Groundwater in the Mezquital Valley, Hidalgo State, Mexico.* México: Comisión Nacional del Agua.

[B15] Broszat M. Nacke H. Blasi R. Siebe C. Huebner J. Daniel R. (2014). Wastewater irrigation increases the abundance of potentially harmful gammaproteobacteria in soils in Mezquital Valley, Mexico. *Appl. Environ. Microbiol.* 80 5282–5291. 10.1128/AEM.01295-14 24951788 PMC4136100

[B16] Calderon F. J. Nielsen D. Acosta-Martinez V. Vigil M. F. Lyon D. (2016). Cover crop and irrigation effects on soil microbial communities and enzymes in semiarid agroecosystems of the central great plains of North America. *Pedosphere* 26 192–205. 10.1016/S1002-0160(15)60034-0

[B17] Callahan B. J. Mcmurdie P. J. Holmes S. P. (2017). Exact sequence variants should replace operational taxonomic units in marker-gene data analysis. *ISME J.* 11 2639–2643. 10.1038/ismej.2017.119 28731476 PMC5702726

[B18] Cannon P. F. Buddie A. G. Bridge P. D. de Neergaard E. Lübeck M. Askar M. M. (2012). Lectera, a new genus of the Plectosphaerellaceae for the legume pathogen Volutella colletotrichoides. *Mycokeys* 3 23–36. 10.3897/mycokeys.3.3065

[B19] Caporaso J. G. Kuczynski J. Stombaugh J. Bittinger K. Bushman F. D. Costello E. K. (2010). QIIME allows analysis of high- throughput community sequencing data. *Nat. Methods* 7 335–336. 10.1038/nmeth0510-335 20383131 PMC3156573

[B20] Chowdhury N. Marschner P. Burns R. (2011). Response of microbial activity and community structure to decreasing soil osmotic and matric potential. *Plant Soil* 344 241–254. 10.1007/s11104-011-0743-9

[B21] Ciccolini V. Bonari E. Pellegrino E. (2015). Land-use intensity and soil properties shape the composition of fungal communities in Mediterranean peaty soils drained for agricultural purposes. *Biol. Fertil. Soils* 51 719–731. 10.1007/s00374-015-1013-4

[B22] Conagua and Semarnat (2015). Estadísticas Agrícolas de los Distritos de Riego. 10.1007/s00374-015-1013-4

[B23] Crowther T. W. Boddy L. Jones T. H. (2012). Functional and ecological consequences of saprotrophic fungus–grazer interactions. *ISME J.* 6 1992–2001. 10.1038/ismej.2012.53 22717883 PMC3475375

[B24] Dalkmann P. Broszat M. Siebe C. Willaschek E. Sakinc T. Huebner J. (2012). Accumulation of pharmaceuticals, *Enterococcus*, and resistance genes in soils irrigated with wastewater for zero to 100 years in central Mexico. *PLoS One* 7:e45397. 10.1371/journal.pone.0045397 23049795 PMC3458031

[B25] de Cáceres M. (2013). *How to use the indicspecies package (ver. 1.7.1). R Proj.*, 29.

[B26] Disciglio G. Gatta G. Libutti A. Gagliardi A. Carlucci A. Lops F. (2015). Effects of irrigation with treated agro-industrial wastewater on soil chemical characteristics and fungal populations during processing tomato crop cycle. *J. Soil Sci. Plant Nutr.* 15 765–780.

[B27] Edgar R. C. (2016). UNOISE2: improved error-correction for Illumina 16S and ITS amplicon sequencing. *bioRxiv*

[B28] Edgar R. C. Haas B. J. Clemente J. C. Quince C. Knight R. (2011). UCHIME improves sensitivity and speed of chimera detection. *Bioinformatics* 27 2194–2200. 10.1093/bioinformatics/btr381 21700674 PMC3150044

[B29] Friedel J. K. Langer T. Siebe C. Stahr K. (2000). Effects of long-term waste water irrigation on soil organic matter, soil microbial biomass and its activities in central Mexico. *Biol. Fertil. Soils* 31 414–421. 10.1007/s003749900188

[B30] García-Orenes F. Caravaca F. Morugán-Coronado A. Roldán A. (2015). Prolonged irrigation with municipal wastewater promotes a persistent and active soil microbial community in a semiarid agroecosystem. *Agric. Water Manag.* 149 115–122. 10.1016/j.agwat.2014.10.030

[B31] Garrido-Jurado I. Fernández-Bravo M. Campos C. Quesada-Moraga E. (2015). Diversity of entomopathogenic Hypocreales in soil and phylloplanes of five Mediterranean cropping systems. *J. Invertebr. Pathol.* 130 97–106. 10.1016/j.jip.2015.06.001 26146223

[B32] Gonçalves Lisboa J. F. Montandon Chaer G. Ferreira Fernandes M. Louro Berbara L. R. Emoke Madari B. (2014). The match between microbial community structure and soil properties is modulated by land use types and sample origin within an integrated agroecosystem. *Soil Biol. Biochem.* 78 97–108. 10.1016/j.soilbio.2014.07.017

[B33] Grube M. Wedin M. (2016). Lichenized fungi and the evolution of symbiotic organization. *Microbiol. Spectr.* 4:FUNK-0011-2016. 10.1128/microbiolspec.FUNK-0011-2016.Correspondence 28087947

[B34] Guarro J. (2013). Fusariosis, a complex infection caused by a high diversity of fungal species refractory to treatment. *Eur. J. Clin. Microbiol. Infect. Dis.* 32 1491–1500. 10.1007/s10096-013-1924-7 23934595

[B35] Hawkes C. V. Kivlin S. N. Rocca J. D. Huguet V. Thomsen M. A. Blake S. K. (2011). Fungal community responses to precipitation. *Glob. Change Biol.* 17 1637–1645. 10.1111/j.1365-2486.2010.02327.x

[B36] He D. Xiang X. He J. Wang C. Cao G. Adams J. (2016). Composition of the soil fungal community is more sensitive to phosphorus than nitrogen addition in the alpine meadow on the Qinghai-Tibetan Plateau. *Biol. Fertil. Soils* 52 1059–1072. 10.1007/s00374-016-1142-4

[B37] Hong C. X. Moorman G. W. (2005). Plant pathogens in irrigation water: challenges and opportunities. *Crit. Rev. Plant Sci.* 24 189–208. 10.1080/07352680591005838

[B38] Hu X. P. Wang M. X. Hu D. F. Yang J. R. (2011). First report of wilt on alfalfa in china caused by *Verticillium nigrescens*. *Plant Dis.* 95:1591. 10.1094/PDIS-07-11-0580 30732015

[B39] Inderbitzin P. Bostock R. M. Davis R. M. Usami T. Platt H. W. Subbarao K. V. (2011). Phylogenetics and taxonomy of the fungal vascular wilt pathogen *Verticillium*, with the descriptions of five new species. *PLoS One* 6:e28341. 10.1371/journal.pone.0028341 22174791 PMC3233568

[B40] IUSS Working Group WRB (2014). *World Reference Base for Soil Resources 2014. International Soil Classification System for Naming Soils and Creating Legends for Soil Maps.* Rome: IUSS Working Group WRB 10.1017/S0014479706394902

[B41] Jansa J. Erb A. Oberholzer H.-R. Smilauer P. Simon E. (2014). Soil and geography are more important determinants of indigenous arbuscular mycorrhizal communities than management practices in Swiss agricultural soils. *Mol. Ecol.* 23 2118–2135. 10.1111/mec.12706 24611988

[B42] Kasel S. Bennett L. T. Tibbits J. (2008). Land use influences soil fungal community composition across central Victoria, south-eastern Australia. *Soil Biol. Biochem.* 40 1724–1732. 10.1016/j.soilbio.2008.02.011

[B43] Kepler R. M. Sung G.-H. Ban S. Nakagiri A. Chen M.-J. Huang B. (2012). New teleomorph combinations in the entomopathogenic genus *Metacordyceps*. *Mycologia* 104 182–197. 10.3852/11-070 22067304

[B44] Klabi R. Bell T. H. Hamel C. Iwaasa A. Schellenberg M. Raies A. (2015). Plant assemblage composition and soil P concentration differentially affect communities of AM and total fungi in a semi-arid grassland. *FEMS Microbiol. Ecol.* 91 1–13. 10.1093/femsec/fiu015 25764537

[B45] Koljalg U. Nilsson R. H. Abarenkov K. Tedersoo L. Taylor A. F. S. Bates S. T. (2013). Towards a unified paradigm for sequence-based identification of fungi. *Mol. Ecol.* 22 5271–5277. 10.1111/mec.12481 24112409

[B46] Koohafkan P. Stewart B. A. (2008). *Water and Cereals in Drylands.* London: The Food and Agriculture Organization of the United Nations and Earthscan.

[B47] Lauber C. L. Hamady M. Knight R. Fierer N. (2009). Pyrosequencing-based assessment of soil pH as a predictor of soil bacterial community structure at the continental scale. *Appl. Environ. Microbiol.* 75 5111–5120. 10.1128/AEM.00335-09 19502440 PMC2725504

[B48] Lauber C. L. Strickland M. S. Bradford M. A. Fierer N. (2008). The influence of soil properties on the structure of bacterial and fungal communities across land-use types. *Soil Biol. Biochem.* 40 2407–2415. 10.1016/j.soilbio.2008.05.021

[B49] Lekberg Y. Vasar M. Bullington L. S. Sepp S.-K. Antunes P. Bunn R. (2018). More bang for the buck? Can arbuscular mycorrhizal fungal communities be characterized adequately alongside other fungi using general fungal primers? *New Phytol.* 220 971–976. 10.1111/nph.15035 29388685

[B50] Ligon J. M. Hill D. S. Hammer P. E. Torkewitz N. R. Hofmann D. Kempf H.-J. (2000). Natural products with antifungal activity from *Pseudomonas* biocontrol bacteria †. *Pest Manag. Sci.* 56 688–695. 10.1002/1526-4998(200008)56

[B51] Lombard L. Van Der Merwe N. A. Groenewald J. Z. Crous P. W. (2015). Generic concepts in *Nectriaceae*. *Stud. Mycol.* 80 189–245. 10.1016/j.simyco.2014.12.002 26955195 PMC4779799

[B52] Lopes Leal P. Siqueira J. O. Stürmer S. L. (2013). Switch of tropical Amazon forest to pasture affects taxonomic composition but not species abundance and diversity of arbuscular mycorrhizal fungal community. *Appl. Soil Ecol.* 71 72–80. 10.1016/j.apsoil.2013.05.010

[B53] Lüneberg K. Schneider D. Siebe C. Daniel R. (2018). Drylands soil bacterial community is affected by land use change and different irrigation practices in the Mezquital Valley, Mexico. *Sci. Rep.* 8:1413. 10.1038/s41598-018-19743-x 29362388 PMC5780513

[B54] Lupatini M. Josemar R. Jacques S. Antoniolli Z. I. Suleiman A. K. A. Fulthorpe R. R. (2013). Land-use change and soil type are drivers of fungal and archaeal communities in the Pampa biome. *World J. Microbiol. Biotechnol.* 29 223–233. 10.1007/s11274-012-1174-3 23054698

[B55] Ma L. Guo C. Lü X. Yuan S. Wang R. (2015). Soil moisture and land use are major determinants of soil microbial community composition and biomass at a regional scale in northeastern China. *Biogeoscience* 12 2585–2596. 10.5194/bg-12-2585-2015

[B56] Maestre F. T. Delgado-Baquerizo M. Jeffries T. C. Eldridge D. J. Ochoa V. (2015). Increasing aridity reduces soil microbial diversity and abundance in global Drylands. *Proc. Natl. Acad. Sci. U.S.A.* 112 15684–15689. 10.1073/pnas.1516684112 26647180 PMC4697385

[B57] Manamgoda D. S. Rossman A. Y. Castlebury L. A. Crous P. W. Madrid H. Chukeatirote E. (2014). The genus *Bipolaris*. *Stud. Mycol.* 79 221–288. 10.1016/j.simyco.2014.10.002 25492990 PMC4255534

[B58] Martin M. (2011). Cutadapt removes adapter sequences from high-throughput sequencing reads. *EMBnet J.* 17 10–12.

[B59] McGuire K. L. D’Angelo H. Brearley F. Q. Gedallovich S. M. Babar N. Yang N. (2015). Responses of soil fungi to logging and oil palm agriculture in southeast Asian tropical forests. *Microb. Ecol.* 69 733–747. 10.1007/s00248-014-0468-4 25149283

[B60] Mchugh T. A. Koch G. W. Schwartz E. (2014). Minor changes in soil bacterial and fungal community composition occur in response to monsoon precipitation in a semiarid grassland. *Soil Microbiol.* 68 370–378. 10.1007/s00248-014-0416-3 24743883

[B61] Miransari M. (2010). Contribution of arbuscular mycorrhizal symbiosis to plant growth under different types of soil stress. *Plant Biol.* 12 563–569. 10.1111/j.1438-8677.2009.00308.x 20636898

[B62] Moora M. Davison J. Öpik M. Metsis M. Saks Ü. Jairus T. (2014). Anthropogenic land use shapes the composition and phylogenetic structure of soil arbuscular mycorrhizal fungal communities. *FEMS Microbiol. Ecol.* 90 609–621. 10.1111/1574-6941.12420 25187481

[B63] Morris E. K. Buscot F. Herbst C. Meiners T. Obermaier E. Wäschke N. W. (2013). Land use and host neighbor identity effects on arbuscular mycorrhizal fungal community composition in focal plant rhizosphere. *Biodivers. Conserv.* 22 2193–2205. 10.1007/s10531-013-0527-z

[B64] Munyanziza E. Kehri H. K. Bagyaraj D. J. (1997). Agricultural intensification, soil biodiversity and agro-ecosystem function in the tropics: the role of mycorrhiza in crops and trees. *Appl. Soil Ecol.* 6 77–85. 10.1016/s0929-1393(96)00152-7

[B65] Nguyen N. H. Song Z. Bates S. T. Branco S. Tedersoo L. Menke J. (2016). FUNGuild: an open annotation tool for parsing fungal community datasets by ecological guild. *Fungal Ecol.* 20 241–248. 10.1016/j.funeco.2015.06.006

[B66] Norton-Brandão D. Scherrenberg S. M. van Lier J. B. (2013). Reclamation of used urban waters for irrigation purposes - A review of treatment technologies. *J. Environ. Manage.* 122 85–98. 10.1016/j.jenvman.2013.03.012 23562951

[B67] Oksanen A. J. Blanchet F. G. Kindt R. Legendre P. Minchin P. R. Hara R. B. O. (2015). *vegan: Community Ecology Package. R Packag. version 2.3-2.*

[B68] Önder D. Aydin M. Berberoglu S. Önder S. Yano T. (2009). The use of aridity index to assess implications of climatic change for land cover in Turkey. *Turk. J. Agric. For.* 33 305–314. 10.3906/tar-0810-21

[B69] Orgiazzi A. Lumini E. Nilsson R. H. Girlanda M. Vizzini A. Bonfante P. (2012). Unravelling soil fungal communities from different Mediterranean land-use backgrounds. *PLoS One* 7:e34847. 10.1371/journal.pone.0034847 22536336 PMC3335027

[B70] Ortega-Larrocea M. P. Siebe C. Bécard G. Méndez I. Webster R. (2001). Impact of a century of wastewater irrigation on the abundance of arbuscular mycorrhizal spores in the soil of the Mezquital Valley of Mexico. *Appl. Soil Ecol.* 16 149–157. 10.1016/s0929-1393(00)00105-0

[B71] Pankhurst C. Yu S. Hawke B. Harch B. (2001). Capacity of fatty acid profiles and substrate utilization patterns to describe differences in soil microbial communities associated with increased salinity or alkalinity at three locations in South Australia. *Biol. Fertil. Soils* 33 204–217. 10.1007/s003740000309

[B72] Porcel R. Aroca R. Ruiz-lozano J. M. (2012). Salinity stress alleviation using arbuscular mycorrhizal fungi. A review. *Agron. Sustain. Dev.* 32 181–200. 10.1007/s13593-011-0029-x

[B73] Porras-Alfaro A. Bayman P. (2011). Hidden fungi, emergent properties: endophytes and microbiomes. *Annu. Rev. Phytopathol.* 49 291–315. 10.1146/annurev-phyto-080508-081831 19400639

[B74] Purahong W. Hoppe B. Kahl T. Schloter M. Schulze E.-D. Bauhus J. (2014). Changes within a single land-use category alter microbial diversity and community structure: molecular evidence from wood-inhabiting fungi in forest ecosystems. *J. Environ. Manage.* 139 109–119. 10.1016/j.jenvman.2014.02.031 24681650

[B75] Qin H. Lu K. Strong P. J. Xu Q. Wu Q. Xu Z. (2015). Long-term fertilizer application effects on the soil, root arbuscular mycorrhizal fungi and community composition in rotation agriculture. *Appl. Soil Ecol.* 89 35–43. 10.1016/j.apsoil.2015.01.008

[B76] R Core Team (2016). *R: A Language and Environment for Statistical Computing*, ed. R Foundation for Statistical Computing (Vienna: R Foundation for Statistical Computing).

[B77] Ramalho da Silva I. Aragão de Mello M. C. Alves Neto F. R. Alves da Silva K. D. Laurênio de Melo A. Oehl F. (2014). Diversity of arbuscular mycorrhizal fungi along an environmental gradient in the Brazilian semiarid. *Appl. Soil Ecol.* 84 166–175. 10.1016/j.apsoil.2014.07.008

[B78] Rath K. M. Rousk J. (2015). Salt effects on the soil microbial decomposer community and their role in organic carbon cycling: a review. *Soil Biol. Biochem.* 81 108–123. 10.1016/j.soilbio.2014.11.001

[B79] Rousk J. Baath E. Brookes P. C. Lauber C. L. Lozupone C. Caporaso J. G. (2010). Soil bacterial and fungal communities across a pH gradient in an arable soil. *ISME J.* 4 1340–1351. 10.1038/ismej.2010.58 20445636

[B80] Rousk J. Brookes P. C. Bååth E. (2009). Contrasting soil pH effects on fungal and bacterial growth suggest functional redundancy in carbon mineralization. *Appl. Environ. Microbiol.* 75 1589–1596. 10.1128/AEM.02775-08 19151179 PMC2655475

[B81] Sánchez-González A. Chapela-Lara M. German-Venegas E. Fuentes-García R. del Ro-Portilla F. Siebe C. (2017). Changes in quality and quantity of soil organic matter stocks resulting from wastewater irrigation in formerly forested land. *Geoderma* 306 99–107. 10.1016/j.geoderma.2017.07.009

[B82] Sanjaya Y. Ocampo V. R. Caoili B. L. (2016). Pathogenicity of three entomopathogenic fungi, *Metarhizium anisopliae*, *Beauveria bassiana*, and *Paecilomyces lilacinus*, to *Tetranychus kanzawai* infesting papaya seedlings. *Arthropods* 5 109–113.

[B83] Sardinha M. Müller T. Schmeisky H. Joergensen R. G. (2003). Microbial performance in soils along a salinity gradient under acidic conditions. *Appl. Soil Ecol.* 23 237–244. 10.1016/S0929-1393(03)00027-1

[B84] Shannon P. Markiel A. Ozier O. Baliga N. S. Wang J. T. Ramage D. (2003). Cytoscape: a software environment for integrated models of biomolecular interaction networks. *Genome Res.* 13 2498–2504. 10.1101/gr.1239303 14597658 PMC403769

[B85] Shrestha K. Stevens S. Shrestha P. Adetutu E. M. Walsh K. B. Ball A. S. (2015). Characterisation of the soil microbial community of cultivated and uncultivated vertisol in Australia under several management regimes. *Agric. Ecosyst. Environ.* 199 418–427. 10.1016/j.agee.2014.10.002

[B86] Siebe C. (1995). Heavy metals availability to plants irrigated with wastewater from Mexico City. *Water Sci. Technol.* 32 29–34. 10.2166/wst.1995.0452

[B87] Siebe C. (1998). Nutrient inputs to soil and their uptake by alfalfa through long-term irrigation with untreated sewage effluent in Mexico. *Soil Use Manag.* 14 119–122. 10.1111/j.1475-2743.1998.tb00628.x

[B88] Siebe C. Cifuentes E. (1995). Environmental impact of wastewater irrigation in central Mexico: an overview. *Int. J. Environ. Health Res.* 5 161–173. 10.1080/09603129509356845

[B89] Siemens J. Huschek G. Siebe C. Kaupenjohann M. (2008). Concentrations and mobility of human pharmaceuticals in the world’s largest wastewater irrigation system, Mexico City-Mezquital Valley. *Water Res.* 42 2124–2134. 10.1016/j.watres.2007.11.019 18083208

[B90] Soon Y. K. Hendershot W. H. (1992). “Soil chemical analysis,” in *Soil Sampling and Methods of Analysis*, eds Carter M. R. Gregorich E. G. (Boca Raton, FL: CRC Press), 173–331.

[B91] Steven B. Gallegos-Graves L. V. Yeager C. Belnap J. Kuske C. R. (2014). Common and distinguishing features of the bacterial and fungal communities in biological soil crusts and shrub root zone soils. *Soil Biol. Biochem.* 69 302–312. 10.1016/j.soilbio.2013.11.008

[B92] Stirling G. R. (2014). *Biological Control of Plant-Parasitic Nematodes. Soil Ecosystem Management in Agricultural Systems*, 2nd Edn. Croydon: CPI Group.

[B93] Tedersoo L. Bahram M. Põlme S. Kõljalg U. Yorou N. S. Wijesundera R. (2014). Global diversity and geography of soil fungi. *Science* 346:1256688. 10.1126/science.aaa1185 25430773

[B94] Tedersoo L. Smith M. E. (2013). Lineages of ectomycorrhizal fungi revisited: foraging strategies and novel lineages revealed by sequences from belowground. *Fungal Biol. Rev.* 27 83–99. 10.1016/j.fbr.2013.09.001

[B95] Toju H. Tanabe A. S. Yamamoto S. Sato H. (2012). High-coverage ITS primers for the DNA-based identification of ascomycetes and basidiomycetes in environmental samples. *PLoS One* 7:e40863. 10.1371/journal.pone.0040863 22808280 PMC3395698

[B96] Treseder K. K. Lennon J. T. (2015). Fungal traits that drive ecosystem dynamics on land. *Microbiol. Mol. Biol. Rev. MMBR* 79 243–262. 10.1128/MMBR.00001-15 25971588 PMC4429240

[B97] United Nations Environmental Management Group [UNEMG] (2011). *Global Drylands: A UN System-Wide Response.* Nairobi: UNEMG.

[B98] van Reeuwijk L. P. (ed.). (2002). *Procedures for Soil Analysis*, 6th Edn. Wageningen: FAO-UN Available at: http://library.wur.nl/WebQuery/clc/546300

[B99] Vargas-Gastélum L. Romero-Olivares A. L. Escalante A. E. Rocha-Olivares A. Brizuela C. Riquelme M. (2015). Impact of seasonal changes on fungal diversity of a semi-arid ecosystem revealed by 454 pyrosequencing. *FEMS Microbiol. Ecol.* 91:fiv044. 10.1093/femsec/fiv044 25877341

[B100] Verbruggen E. Xiang D. Chen B. Xu T. Rillig M. C. (2015). Mycorrhizal fungi associated with high soil N:P ratios are more likely to be lost upon conversion from grasslands to arable agriculture. *Soil Biol. Biochem.* 86 1–4. 10.1016/j.soilbio.2015.03.008

[B101] Wang Z. Chen Q. Liu L. Wen X. Liao Y. (2016). Responses of soil fungi to 5-year conservation tillage treatments in the Drylands of Northern China. *Appl. Soil Ecol.* 101 132–140. 10.1016/j.apsoil.2016.02.002

[B102] White T. J. Bruns T. D. Lee S. B. Taylor J. W. (1990). “Amplification and direct sequencing of fungal ribosomal RNA genes for phylogenetics,” in *PCR Protocols: A Guide to Methods and Applications*, eds Innis M. A. Gelfand D. H. Sninsky J. J. White T. J. (New York, NY: Academic Press Inc), 315–322. 10.1016/b978-0-12-372180-8.50042-1

[B103] Xiang D. Verbruggen E. Hu Y. Veresoglou S. D. Rillig M. C. Zhou W. (2014). Land use influences arbuscular mycorrhizal fungal communities in the farming–pastoral ecotone of northern China. *New Phytol.* 204 968–978. 10.1111/nph.12961 25103342

[B104] Xiao M. Wang H. Lu J. Chen S. C.-A. Kong F. Ma X.-J. (2014). Three clustered cases of candidemia caused by *Candida quercitrusa* and mycological characteristics of this novel species. *J. Clin. Microbiol.* 52 3044–3048. 10.1128/JCM.00246-14 24696025 PMC4136143

[B105] Yang G. Liu N. Lu W. Wang S. Kan H. Zhang Y. (2014). The interaction between arbuscular mycorrhizal fungi and soil phosphorus availability in fl uences plant community productivity and ecosystem stability. *J. Ecol.* 102 1072–1082. 10.1111/1365-2745.12249

[B106] Yang T. Adams J. M. Shi Y. He J. Jing X. Chen L. (2017). Soil fungal diversity in natural grasslands of the Tibetan Plateau: associations with plant diversity and productivity. *New Phytol.* 215 756–765. 10.1111/nph.14606 28542845

[B107] Zhang J. Kobert K. Flouri T. Stamatakis A. (2014). PEAR: a fast and accurate Illumina Paired-End reAd mergeR. *Bioinformatics* 30 614–620. 10.1093/bioinformatics/btt593 24142950 PMC3933873

[B108] Zheng Y. Hu H.-W. Guo L.-D. Anderson I. C. Powell J. R. (2017). Dryland forest management alters fungal community composition and decouples assembly of root- and soil-associated fungal communities. *Soil Biol. Biochem.* 109 14–22. 10.1016/j.soilbio.2017.01.024

