# Supplementary material for: Land Use Change and Water Quality Use for Irrigation Alters Drylands Soil Fungal Community in the Mezquital Valley, Mexico
Source: Front Microbiol. 2019 Jun 14;10:1220. doi: 10.3389/fmicb.2019.01220 (PMC6587704; doi:10.3389/fmicb.2019.01220)
Supplement: Supplementary file 1 [file Data_Sheet_1.pdf]

Drylands soil fungal community is affected by land use change and different irrigation practices in the Mezquital Valley, Mexico

Kathia Lüneberg[1*], Dominik Schneider[2], Nicole Brinkmann[3], Christina Siebe[1], Rolf Daniel[2]

Supplementary material

Content

Text S1. Study sites and sample collection.

Text S2. Characterization of soil parameters.

Figure S1. Location of the study sites within the Mezquital Valley (Mexico).

Figure S2. Soil properties.

Figure S3. Rarefaction curves of the fungal dataset.

Figure S4. Relative abundance of phyla that presented significant differences among land use systems.

Figure S5. Top 19 most abundant identified saprothrophic genera.

Figure S6. Top 7 most abundant symbiothophic genera.

Figure S7. Top 20 most abundant patothophic genera.

Figure S8. Relative abundance of human pathogenic fungi in soil.

Table S1. Spearman correlation coefficient and variance inflation factors (VIF) of soil properties.

Table S2. Spearman correlation coefficient between fungal families associated to each land use system and soil properties (provided as a separate pdf file).

Table S3. Relative abundance of pathogenic fungi in wastewater samples.

Text S1. Study sites and sample collection

The natural vegetation in the area is classified as xerophytic shrubs with mesquite (*Prosopis juliflora*) as the dominant tree species. Shrubland areas are located in the mountains and upper piedmont areas, where no irrigation infrastructure exists. The main crop is maize (*Zea mays*), which is produced in the rainy season (June-October), or alfalfa (*Medicago sativa*), a perennial fodder crop under irrigated agriculture. Alfalfa is rotated with maize (three years of alfalfa and two years of maize). The fields under rainfed agriculture are abandoned the rest of the year. The lowest areas of the valley are irrigated periodically (every 30 days on average) with fresh water pumped from deep wells, untreated wastewater coming from Mexico City or wastewater temporally stored in the Endhó dam where a sedimentation process occurs during 3 months of storage. Irrigation is performed by flooding, each irrigation with 200 mm on average (BGS, 1998). Alfalfa receives 10 irrigations per year and maize 6 irrigations during the growing cycle (Lüneberg et al., 2018).

Text S2. Characterization of soil parameters

The soil of all the plots classify as Haplic Phaeozem (IUSS, 2014) with clay to silty clay loam texture. The soil properties among land use systems differed significantly and some properties such as moisture, electrical conductivity and P content also differed between seasons (Table 1; Supplementary material Fig. S2). Soil moisture was higher in soils irrigated with DWW and UTWW with more than 30%, while the rest of the land use systems had less than 19%. Rainfed and FW showed higher moisture during the rainy season. The pH ranged from 7.1 to 7.8, being the lowest in shrubland and the highest in rainfed and FW soil. In the rainfed soil, N and C content was lowest compared to the other systems. Several soil properties increased from rainfed to UTWW, such as electrical conductivity, and the amount of sodium, potassium, magnesium and phosphorous.

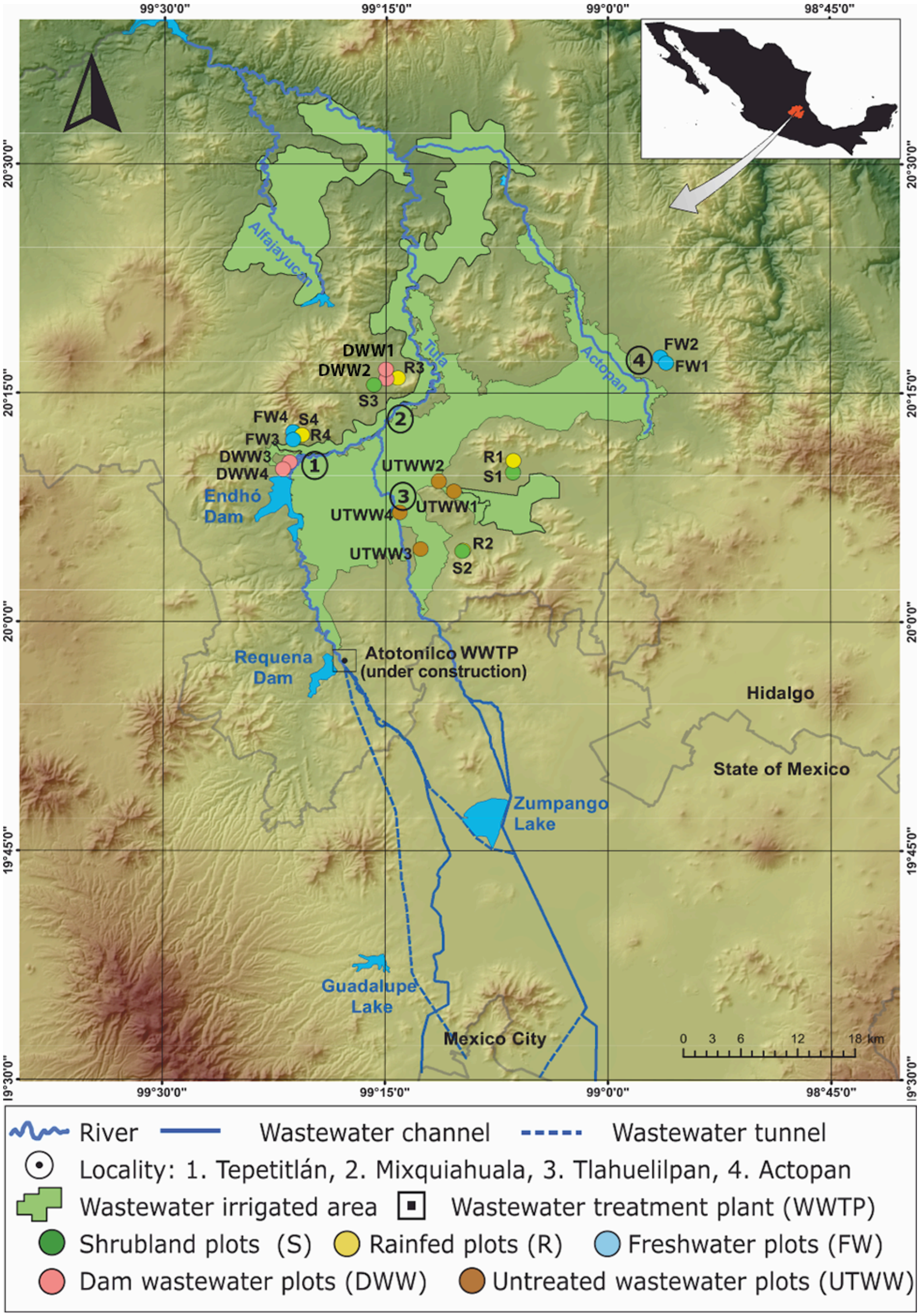

Figure S1. Location of the study sites within the Mezquital Valley (Mexico) (Lüneberg et al., 2018). Map was created with ESRI 2011. ArcGIS Desktop: Release 10. Redlands, CA: Environmental Systems Research Institute.

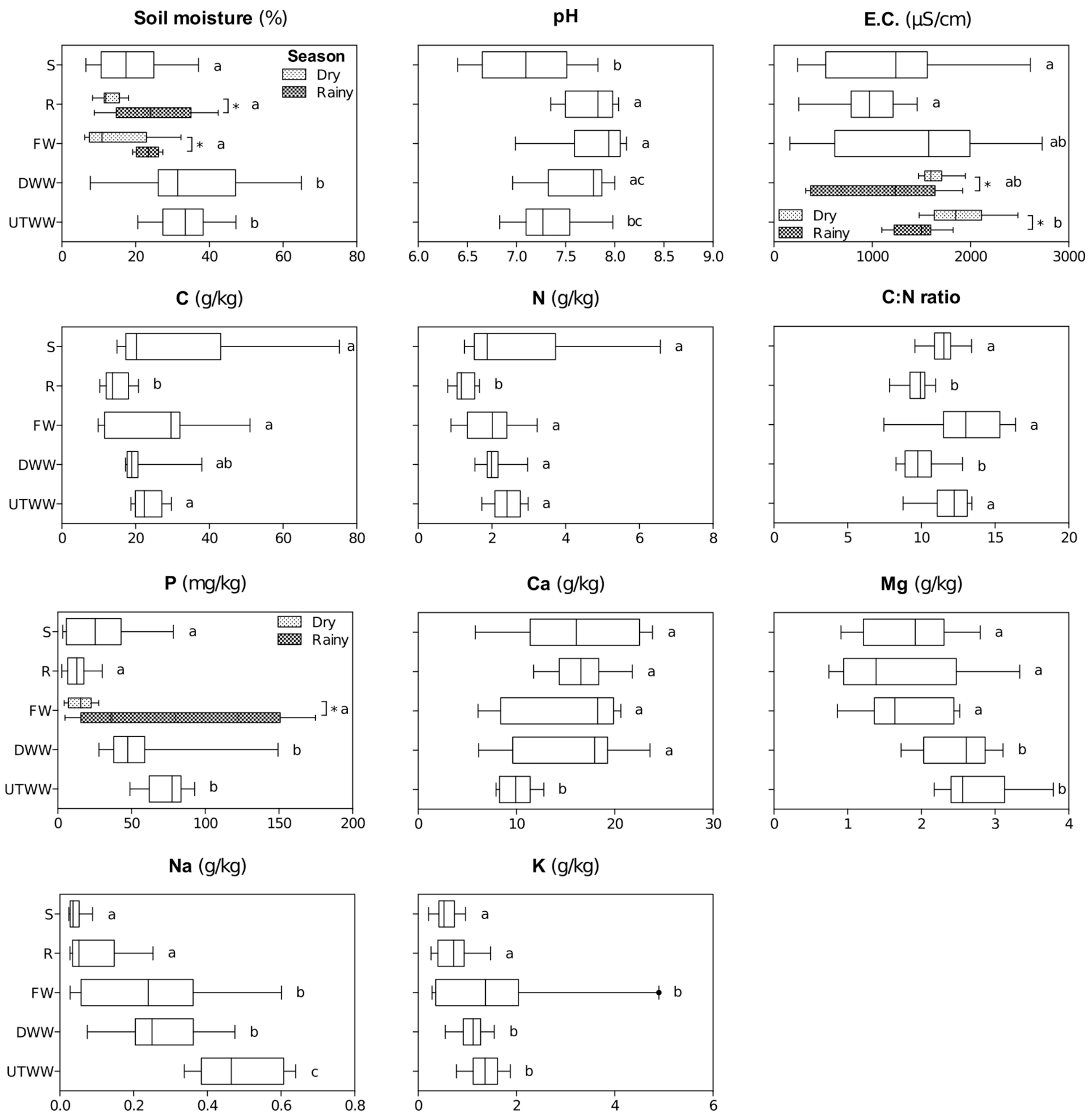

Figure S2. Soil properties in Shrubland (S), Rainfed (R), Freshwater (FW), Dam wastewater (DWW) and Untreated wastewater (UTWW) systems, during the dry and rainy season. Box are extended from the 25th to 75th percentiles, the line in the box is plotted at the median. Whiskers represent the smallest and the largest value. Kruskal-Wallis and Dunn's tests were used to determined differences among land use systems, and permutation test for differences between seasons. Different letters indicate statistical difference among land use systems and * indicate statistical differences between seasons ($p \leq 0.05$). Only parameters differing significantly between seasons in each land use system are shown, if they did not differ, samples of both seasons were merged (Lüneberg et al., 2018).

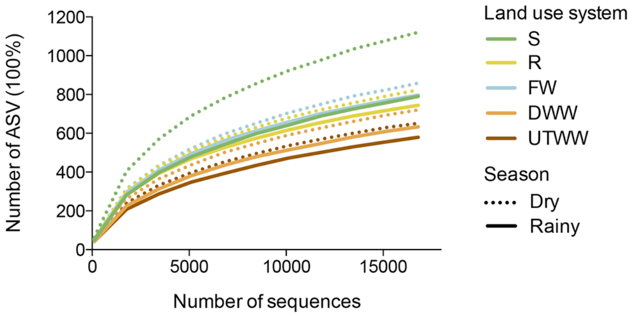

Figure S3. Rarefaction curves of the fungal dataset in Shrubland (S), Rainfed (R), Freshwater (FW), Dam wastewater (DWW) and Untreated wastewater (UTWW) systems, during the dry and rainy season.

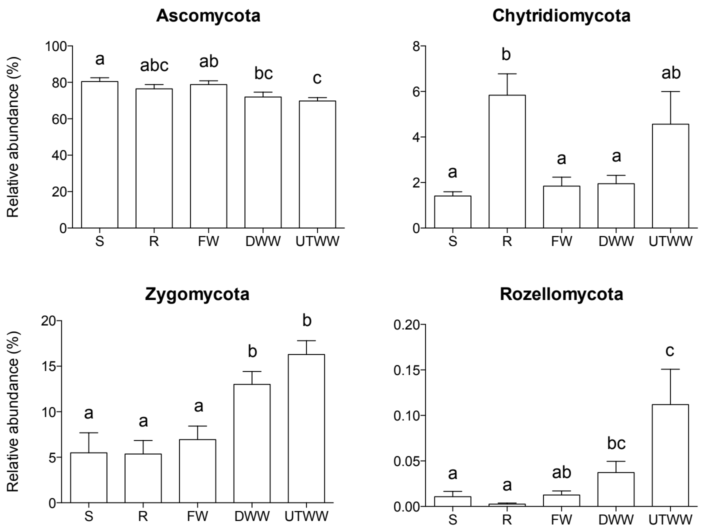

Figure S4. Relative abundance of phyla that presented significant differences among land use systems. Shrubland (S), Rainfed (R), Freshwater (FW), Dam wastewater (DWW) and Untreated wastewater (UTWW). Kruskal-Wallis and Dunn's tests were used to determined differences among land use systems. Different letters indicate statistical significance among land use systems.

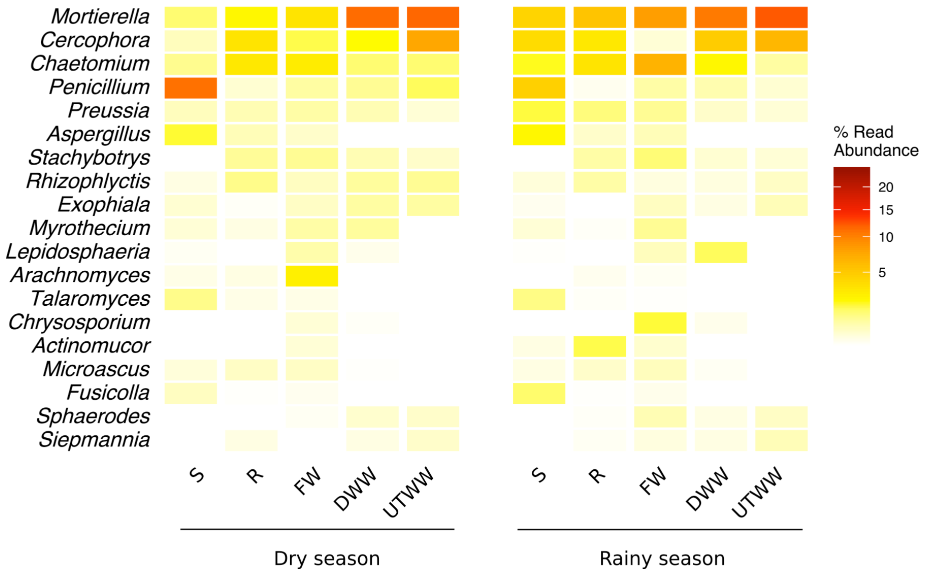

Figure S5. Top 19 most abundant identified saprothrophic genera. Land use systems: Shrubland (S), Rainfed (R), Freshwater (FW), Dam wastewater (DWW) and Untreated wastewater (UTWW) irrigated, during dry and rainy season.

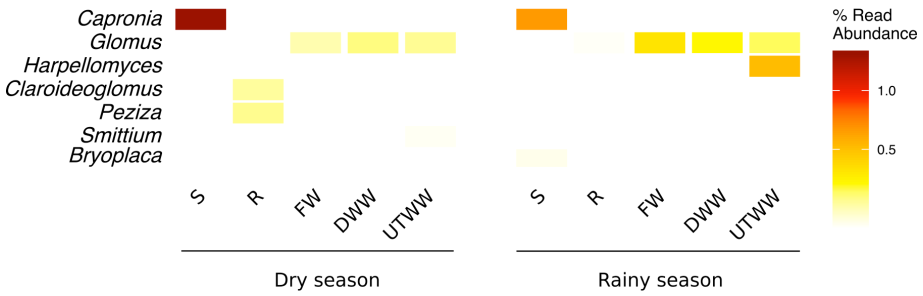

Figure S6. Top 7 most abundant symbiothophic genera. Land use systems: Shrubland (S), Rainfed (R), Freshwater (FW), Dam wastewater (DWW) and Untreated wastewater (UTWW) irrigated, during dry and rainy season.

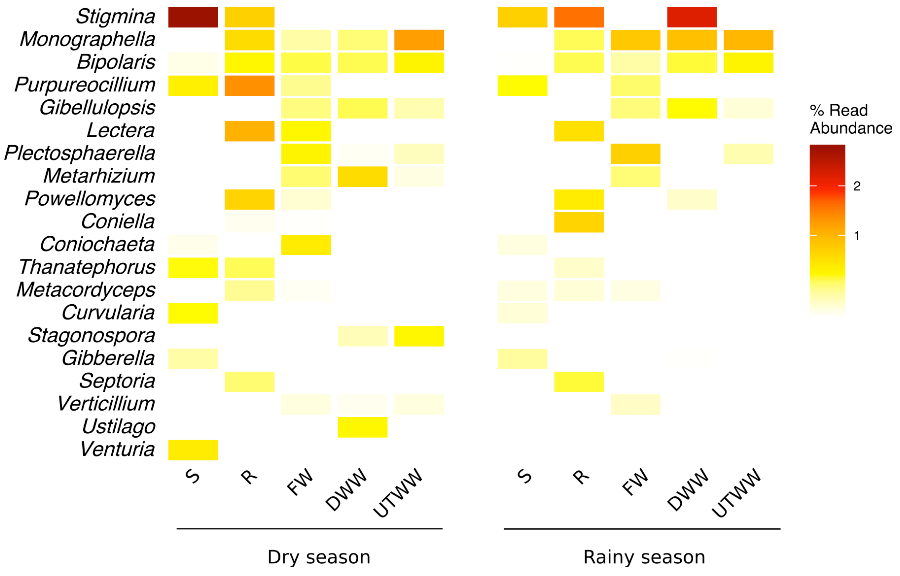

Figure S7. Top 20 most abundant patothophic genera. Land use systems: Shrubland (S), Rainfed (R), Freshwater (FW), Dam wastewater (DWW) and Untreated wastewater (UTWW) irrigated, during dry and rainy season.

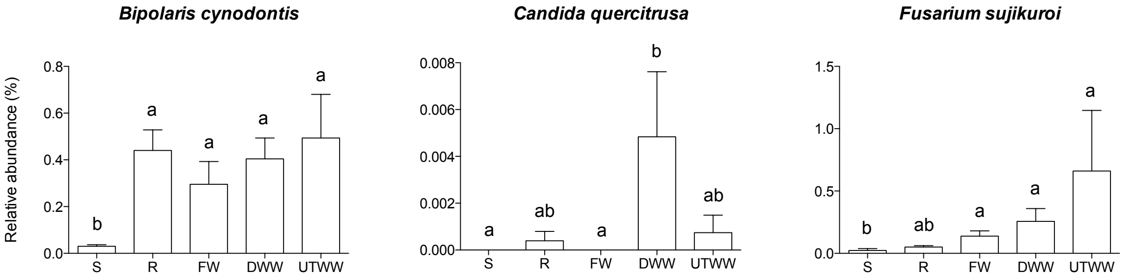

Figure S8. Relative abundance of human pathogenic fungi in soil. Shrubland (S), Rainfed (R), Freshwater (FW), Dam wastewater (DWW) and Untreated wastewater (UTWW) systems. Kruskal-Wallis and Dunn's tests were used to determined differences among land use systems. Different letters indicate statistical significance among land use systems.

Table S1. Spearman correlation coefficient and variance inflation factors (VIF) of soil properties.

	Moisture	pH	E.C.	C	N	C:N	P	Ca	Mg	K	Na	VIF
Moisture		0.05	0.27	0.06	0.13	-0.17	0.29	-0.02	0.57	0.25	0.52	1.77
pH	0.05		0.21	-0.12	-0.29	-0.06	-0.07	0.54	-0.02	0.32	0.10	2.23
E.C.	0.27	0.21		0.37	0.50	0.04	0.43	0.30	0.54	0.63	0.53	3.57
C	0.06	-0.12	0.37		0.80	0.14	0.25	0.23	0.20	0.17	0.05	3.27
N	0.13	-0.29	0.50	0.80		0.18	0.35	0.05	0.29	0.18	0.12	4.76
C:N	-0.17	-0.06	0.04	0.14	0.18		0.06	-0.19	-0.05	0.18	0.13	1.31
P	0.29	-0.07	0.43	0.25	0.35	0.06		-0.17	0.42	0.59	0.48	2.17
Ca	-0.02	0.54	0.30	0.23	0.05	-0.19	-0.17		0.14	0.15	-0.15	2.60
Mg	0.57	-0.02	0.54	0.20	0.29	-0.05	0.42	0.14		0.40	0.64	2.70
K	0.25	0.32	0.63	0.17	0.18	0.18	0.59	0.15	0.40		0.53	2.85
Na	0.52	0.10	0.53	0.05	0.12	0.13	0.48	-0.15	0.64	0.53		3.08

E.C, electrical conductivity; P, available Phosphorous; Ca, Calcium; Mg, Magnesium; K, Potassium; Na, Sodium.

Supplementary material

Table S2. Spearman correlation coefficient between fungal families associated to each land use system and soil properties

	Humidity	pH	E.C.	C	N	P	Na
**Shrubland**							
Archaeorhizomycetaceae	-0.123	**-0.328**	-0.05	0.094	0.034	**-0.308**	**-0.307**
Hysteriaceae	-0.191	**-0.266**	**-0.229**	0.072	0.011	**-0.352**	**-0.466**
Capnodiaceae	-0.092	-0.14	0.203	**0.378**	**0.343**	-0.124	**-0.3**
Patellariaceae	-0.205	**-0.366**	-0.217	0.045	0.017	-0.148	**-0.329**
Dacampiaceae	**-0.321**	**-0.257**	**-0.322**	-0.099	-0.129	**-0.408**	**-0.377**
Didymosphaeriaceae	**-0.236**	**-0.413**	-0.158	0.052	0.032	**-0.267**	**-0.3**
Lentitheciaceae	**-0.294**	**-0.339**	-0.055	0.186	0.073	**-0.269**	**-0.419**
Melanommataceae	**-0.339**	**-0.328**	**-0.317**	-0.1	-0.129	-0.21	**-0.438**
Montagnulaceae	-0.165	**-0.422**	-0.136	0.147	0.186	-0.173	**-0.339**
Sympoventuriaceae	**-0.265**	**-0.264**	**-0.282**	-0.191	-0.196	-0.011	**-0.228**
Venturiaceae	-0.173	**-0.456**	-0.002	0.173	0.214	-0.048	**-0.304**
Chaetothyriaceae	**-0.347**	**-0.306**	0.042	**0.237**	0.088	**-0.225**	**-0.357**
Herpotrichiellaceae	-0.172	**-0.335**	0.17	**0.508**	**0.459**	**0.26**	-0.125
Trichocomaceae	**-0.28**	**-0.23**	-0.107	0.062	0.043	**-0.315**	**-0.497**
Gymnoascaceae	**-0.46**	0.052	**-0.384**	-0.214	**-0.318**	**-0.468**	**-0.548**
Massariaceae	**-0.284**	**-0.456**	-0.168	**0.238**	0.179	-0.217	**-0.532**
Verrucariaceae	-0.05	**-0.308**	0.0004	**0.293**	**0.246**	**0.235**	-0.009
Teloschistaceae	**-0.392**	**-0.384**	**-0.347**	-0.137	-0.172	**-0.518**	**-0.433**
Helotiaceae	**-0.347**	**-0.439**	-0.081	0.159	0.214	-0.171	**-0.378**
Rutstroemiaceae	**-0.377**	-0.108	-0.085	-0.027	-0.158	**-0.361**	**-0.309**
Myxotrichaceae	-0.138	0.115	-0.048	-0.095	-0.122	-0.105	**-0.228**
Thelebolaceae	-0.145	**-0.391**	-0.113	0.187	0.151	-0.057	**-0.363**
Orbiliaceae	-0.025	**-0.376**	**0.234**	0.134	**0.28**	0.18	0.055
Trichomonascaceae	-0.147	-0.096	-0.156	-0.048	-0.171	-0.205	**-0.42**
Togniniaceae	**-0.299**	**-0.358**	-0.202	0.008	-0.031	-0.182	**-0.352**
Valsaceae	-0.168	-0.15	0.005	0.032	0.037	-0.196	**-0.295**
Annulatascaceae	**-0.247**	**-0.439**	-0.205	0.128	0.072	-0.202	**-0.471**
Amphisphaeriaceae	**-0.251**	-0.06	-0.155	-0.023	-0.144	**-0.392**	**-0.312**
Taphrinaceae	0.022	-0.147	0.075	0.209	0.158	-0.002	-0.153

	Humidity	pH	E.C.	C	N	P	Na
Agaricaceae	-0.017	**-0.555**	-0.177	0.012	0.171	-0.068	**-0.298**
Entolomataceae	**-0.454**	-0.16	-0.14	0.042	-0.05	**-0.338**	**-0.418**
Marasmiaceae	**-0.291**	**-0.264**	-0.003	**0.297**	0.151	-0.116	**-0.419**
Tricholomataceae	**-0.383**	-0.08	**-0.252**	-0.018	-0.057	**-0.285**	**-0.537**
Boletaceae	**-0.332**	**-0.4**	-0.094	0.105	0.15	-0.194	**-0.3**
Geastraceae	**-0.555**	-0.213	**-0.412**	-0.174	-0.178	**-0.477**	**-0.707**
Schizoporaceae	-0.044	**-0.223**	0.163	**0.311**	**0.324**	-0.106	**-0.292**
Xenasmataceae	**-0.247**	**-0.388**	0.089	**0.336**	**0.338**	-0.009	**-0.399**
Sebacinaceae	**-0.236**	-0.172	-0.133	-0.014	-0.003	**-0.295**	**-0.357**
Hydnodontaceae	**-0.222**	**-0.259**	**-0.238**	0.013	-0.028	**-0.244**	**-0.429**
Piskurozymaceae	-0.143	**-0.329**	0.002	**0.336**	**0.349**	-0.083	**-0.41**
Endogonaceae	**-0.354**	**-0.38**	-0.179	-0.079	-0.105	**-0.291**	**-0.411**
Cunninghamellaceae	**-0.421**	-0.021	**-0.313**	0.034	-0.138	**-0.508**	**-0.589**
**Rainfed**							
Pleosporaceae	-0.07	0.197	0.099	-0.21	**-0.279**	**-0.308**	0.161
Pezizaceae	**-0.466**	0.141	**-0.233**	-0.187	**-0.398**	**-0.442**	**-0.435**
Pyronemataceae	**-0.249**	0.122	-0.065	**-0.291**	**-0.231**	**-0.339**	**-0.324**
Saccharomycetaceae	-0.012	-0.165	-0.049	-0.217	-0.047	-0.1	0.163
Schizoparmaceae	**-0.384**	**-0.234**	**-0.416**	**-0.308**	**-0.291**	**-0.437**	**-0.533**
Ophiostomataceae	-0.045	0.181	-0.209	**-0.314**	**-0.353**	**-0.251**	-0.143
Hyponectriaceae	**-0.275**	-0.131	**-0.47**	**-0.391**	**-0.378**	**-0.568**	**-0.616**
Hymenochaetaceae	-0.216	-0.064	**-0.377**	**-0.448**	**-0.505**	**-0.413**	**-0.256**
Stephanosporaceae	-0.179	**-0.314**	-0.175	-0.022	-0.06	-0.058	**-0.318**
Filobasidiaceae	**-0.305**	0.19	-0.128	**-0.374**	**-0.419**	**-0.529**	-0.202
Geminibasidiaceae	0.019	0.169	**-0.33**	**-0.553**	**-0.459**	**-0.333**	-0.09
Lobulomycetaceae	0.081	0.15	-0.04	-0.033	0.054	-0.121	-0.093
Spizellomycetaceae	**-0.408**	-0.054	**-0.237**	**-0.258**	**-0.417**	**-0.411**	**-0.265**
Archaeosporaceae	-0.077	**0.258**	-0.068	-0.142	-0.145	0.017	0.019
Claroideoglomeraceae	**-0.49**	0.138	-0.167	-0.216	**-0.338**	**-0.578**	**-0.364**
Kickxellaceae	**-0.578**	0.038	**-0.302**	**-0.291**	**-0.394**	**-0.609**	**-0.419**
Saksenaeaceae	**-0.256**	-0.061	**-0.316**	**-0.275**	**-0.354**	**-0.286**	**-0.381**
**Rainfed + Temporal**							
Parmeliaceae	**-0.277**	**-0.241**	-0.001	0.071	0.141	**-0.242**	**-0.297**
Ceratobasidiaceae	**-0.529**	-0.101	-0.166	**-0.336**	**-0.315**	**-0.542**	**-0.465**

	Humidity	pH	E.C.	C	N	P	Na
Bulleraceae	**-0.321**	**-0.271**	-0.115	-0.149	-0.15	**-0.388**	**-0.309**
**FW**							
Arachnomycetaceae	**-0.416**	**0.238**	**-0.223**	-0.145	**-0.285**	**-0.42**	**-0.36**
Onygenaceae	0.075	**0.461**	**0.287**	**0.236**	0.121	**0.256**	0.099
Hypocreaceae	-0.156	0.205	**0.332**	0.095	0.186	0.103	0.041
Plectosphaerellaceae	0.023	**0.466**	0.146	-0.094	-0.115	0.113	0.216
Strophariaceae	-0.069	-0.009	0.034	0.066	0.095	0.189	0.068
Thelephoraceae	**-0.387**	-0.105	**-0.267**	-0.156	-0.171	**-0.373**	**-0.288**
Buckleyzymaceae	-0.006	**0.253**	0.019	0.005	-0.041	**-0.233**	0.048
**Rainfedl + FW**							
Dothioraceae	**-0.534**	-0.145	**-0.324**	**-0.299**	**-0.289**	**-0.567**	**-0.484**
Sporormiaceae	**-0.458**	0.022	**-0.438**	**-0.255**	**-0.349**	**-0.412**	**-0.653**
Tubeufiaceae	**-0.372**	-0.153	**-0.501**	**-0.338**	**-0.337**	**-0.559**	**-0.69**
Ajellomycetaceae	**-0.341**	0.042	-0.005	0.12	-0.049	**-0.255**	**-0.459**
Arthrodermataceae	**-0.499**	**0.227**	-0.215	0.008	-0.201	**-0.304**	**-0.379**
Microascaceae	**-0.306**	**0.427**	0.03	0.012	-0.143	**-0.258**	**-0.279**
Chaetomiaceae	-0.133	**0.492**	-0.078	-0.103	**-0.318**	-0.212	-0.17
Rhizopogonaceae	-0.193	**0.333**	-0.18	-0.212	**-0.314**	**-0.411**	**-0.271**
Russulaceae	-0.143	0.128	**-0.273**	**-0.301**	**-0.289**	**-0.305**	-0.125
Erratomycetaceae	-0.195	0.195	-0.095	-0.09	**-0.246**	-0.091	**-0.235**
Phakopsoraceae	**-0.47**	0.127	-0.14	-0.175	**-0.279**	**-0.455**	-0.192
Glomosporiaceae	**-0.421**	0.091	**-0.224**	**-0.386**	**-0.418**	**-0.409**	**-0.314**
**FW + DWW +UTWW**							
Cucurbitariaceae	**0.374**	0.082	**0.501**	**0.376**	**0.436**	**0.581**	**0.576**
Ceratostomataceae	**0.263**	**0.267**	**0.44**	0.174	0.176	**0.539**	**0.625**
Cantharellaceae	0.214	0.102	0.152	0.028	0.031	0.167	**0.372**
Gigasporaceae	0.024	-0.142	-0.075	**-0.223**	-0.067	-0.099	0.131
Pilobolaceae	0.039	**0.259**	0.019	-0.139	-0.138	-0.067	0.025
**DWW**							
Aplosporellaceae	0.107	**-0.351**	0.048	0.097	**0.221**	0.17	-0.128
Tephromelataceae	**0.303**	0.08	0.12	-0.036	0.157	**0.295**	**0.315**
Erysiphaceae	**0.298**	0.183	0.118	-0.149	-0.03	-0.065	0.152
Neolectaceae	0.135	0.002	0.106	-0.06	0.074	0.072	0.125
Nectriaceae	**0.362**	0.102	0.049	0.165	**0.272**	**0.361**	**0.308**

	Humidity	pH	E.C.	C	N	P	Na
Mycenaceae	-0.053	-0.023	0.094	0.06	0.16	**0.232**	0.131
Gloeophyllaceae	**0.348**	-0.07	0.17	-0.054	0.142	0.167	0.198
Microbotryaceae	0.133	-0.05	-0.033	0.042	0.124	**0.258**	0.134
Ustilaginaceae	-0.109	-0.01	-0.052	**-0.288**	-0.134	0.091	0.039
Blastocladiaceae	**0.334**	-0.182	**0.255**	0.206	**0.289**	0.201	**0.246**
**UTWW**							
Pichiaceae	**0.33**	-0.141	0.219	0.152	**0.221**	**0.481**	**0.426**
Lasiosphaeriaceae	**0.281**	-0.203	-0.136	-0.045	-0.033	0.194	**0.303**
Heterogastridiaceae	**0.462**	-0.214	0.064	0.081	0.201	**0.456**	**0.459**
Trichosporonaceae	**0.441**	**-0.231**	0.175	-0.005	0.138	**0.374**	**0.498**
Chytridiaceae	0.205	**0.287**	**0.36**	0.147	0.073	**0.364**	**0.442**
Olpidiaceae	0.118	0.185	**0.643**	**0.308**	**0.358**	**0.395**	**0.491**
Sonoraphlyctidaceae	**0.319**	**-0.491**	0.177	0.195	**0.321**	**0.363**	0.195
Harpellaceae	0.200	-0.019	**0.315**	0.167	0.157	**0.478**	**0.528**
Legeriomycetaceae	**0.245**	**-0.287**	0.125	0.015	0.155	**0.43**	**0.259**
**DWW + UTWW**							
Massarinaceae	**0.293**	-0.084	**0.364**	0.192	**0.292**	**0.395**	**0.439**
Vibrisseaceae	**0.325**	0.01	0.219	0.076	**0.272**	**0.484**	**0.44**
Cystobasidiaceae	0.204	**-0.232**	0.166	0.124	0.22	**0.282**	**0.272**
Mortierellaceae	**0.643**	0.067	**0.302**	0.154	**0.247**	**0.53**	**0.55**

FW: freshwater irrigation
DWW: dam wastewater irrigation
UTWW: Untreated wastewater irrigation
Bold numbers indicate significance ($p \leq 0.05$).
E.C, electrical conductivity; P, available Phosphorous; C, carbon, N, nitrogen, Na, Sodium.

Table S3. Relative abundance of pathogenic fungi in wastewater samples.

	DWW	UTWW
Aspergillus flavus	0.032	0.018
Aspergillus fumigatus	0.003	0.026
Candida albicans	0.006	0.047
Candida glabrata	0.059	0.482
Candida tropicalis	0.047	0.167
Cladosporium sphaerospermum	0.002	0.018
Cryptococcus laurentii	0.015	0.006
Malassezia globosa	0.006	0.012
Malassezia restricta	0.006	0.009
Mucor circinelloides	0.012	0.041
Mucor hiemalis	0	0.012
Mucor racemosus	0.056	0.123
Rhizopus arrhizus	0	0.006
Rhodotorula mucilaginosa	0.159	0.679

Dam wastewater (DWW) and Untreated wastewater (UTWW)
